# The role of *Leishmania* GP63 in the modulation of innate inflammatory response to *Leishmania major* infection

**DOI:** 10.1371/journal.pone.0262158

**Published:** 2021-12-31

**Authors:** Aretha Chan, Jose-Mauricio Ayala, Fernando Alvarez, Ciriaco Piccirillo, George Dong, David Langlais, Martin Olivier

**Affiliations:** 1 Department of Microbiology and Immunology, McGill University, Montréal, QC, Canada; 2 Infectious Diseases and Immunity in Global Health Program, Research Institute of the McGill University Health Centre, Montréal, QC, Canada; 3 Department of Human Genetics, McGill Genome Centre, Montréal, QC, Canada; 4 McGill Research Centre on Complex Traits, Montreal, QC, Canada; 5 FOCiS Centre of Excellence in Translational Immunology, Montréal, QC, Canada; Instituto Oswaldo Cruz, BRAZIL

## Abstract

Leishmaniasis is a disease caused by the protozoan parasite *Leishmania* and is known to affect millions of individuals worldwide. In recent years, we have established the critical role played by *Leishmania* zinc-metalloprotease GP63 in the modulation of host macrophage signalling and functions, favouring its survival and progression within its host. *Leishmania major* lacking GP63 was reported to cause limited infection in mice, however, it is still unclear how GP63 may influence the innate inflammatory response and parasite survival in an in vivo context. Therefore, we were interested in analyzing the early innate inflammatory events upon *Leishmania* inoculation within mice and establish whether *Leishmania* GP63 influences this initial inflammatory response. Experimentally, *L*. *major* WT (*L*. *major*^WT^), *L*. *major* GP63 knockout (*L*. *major*^KO^), or *L*. *major* GP63 rescue (*L*. *major*^R^) were intraperitoneally inoculated in mice and the inflammatory cells recruited were characterized microscopically and by flow cytometry (number and cell type), and their infection determined. Pro-inflammatory markers such as cytokines, chemokines, and extracellular vesicles (EVs, e.g. exosomes) were monitored and proteomic analysis was performed on exosome contents. Data obtained from this study suggest that *Leishmania* GP63 does not significantly influence the pathogen-induced inflammatory cell recruitment, but rather their activation status and effector function. Concordantly, internalization of promastigotes during early infection could be influenced by GP63 as fewer *L*. *major*^KO^ amastigotes were found within host cells and appear to maintain in host cells over time. Collectively this study provides a clear analysis of innate inflammatory events occurring during *L*. *major* infection and further establish the prominent role of the virulence factor GP63 to provide favourable conditions for host cell infection.

## Introduction

The host immune response is a significant determinant in the outcome of the disease leishmaniasis. In humans, the two types of disease progression are seen, one where it leads to spontaneous healing, and one where it causes chronic non-healing lesions [1]. Many mice studies indicate that the adaptive immune response is responsible for the outcome of disease [2, 3]. But ultimately, it is the ability of the *Leishmania* to infect cells that determine whether or not disease will manifest. This is dependent on the host’s early innate immune response, and how the parasite can evade or exploit it. *Leishmania* express a variety of virulence factors that mediate the initial interaction between the parasite and the host cell to promote infection [4].

Involved in the receptor-mediated uptake of *Leishmania* and resistance to host defence is an important metalloprotease known as GP63. GP63 is expressed on all studied *Leishmania* species [5] and can degrade a wide range of substrates. Studies report that *Leishmania* GP63 directly cleaves complement C3 to escape complement mediated lysis, whereby the product C3bi can interact with macrophage receptor CR3 to promote attachment and uptake [6]. Past research from our laboratory demonstrated that GP63 is directly involved in altering host macrophage signalling for parasite escape. The known inhibited pathways include protein kinase C signalling pathway, the IFN- γ mediated JAK/STAT, and direct interference with transcription factors NF-κB, STAT1, and AP-1, as well as the induction of negative signalling regulators such as protein tyrosine phosphatase SHP-1 [7]. These inhibitory actions prevent macrophage effector functions from killing *Leishmania*, allowing them to survive within host cells and recruit more cells to further propagate the infection.

In relation to this GP63 mediated action, studies reported that *L*. *major* knockouts for GP63 (*L*. *major*^KO^) were generating a reduced development of cutaneous lesion when inoculated to mice compared to wild type *L*. *major* (*L*. *major*^wt^) [8, 9]. It has been proposed that *Leishmania* lacking GP63 were more sensitive in vitro to complement mediated lysis, which could influence the setting of the infection in vivo. However, the full early innate immune response to *Leishmania* GP63 has not yet been studied in-depth and could provide a better understanding to what extent GP63 influences this initial host response concurring to the establishment of the infection.

Past studies from our laboratory and others have also investigated the modulation of proinflammatory mediators by *Leishmania* [10, 11]. In cutaneous leishmaniasis, lesions form due to chronic inflammation and cell infiltration to the skin. *L*. *major* inoculation to the skin induced the recruitment of leukocytes, which is crucial to both host defence and pathology. It is known that various proinflammatory mediators such as cytokines and chemokines can be rapidly induced upon *L*. *major* infection both in vitro and in vivo to attract inflammatory cells to the site of the infection and therefore offering a greater number of cells to be infected [10]. Furthermore, novel players have been identified and seen to be involved in the inflammatory process [12]; the extracellular vesicles including exosomes are important players in the communication between eukaryotic cells. We have reported that exosomes released by *Leishmania* infected macrophages were found to differ in protein content from uninfected ones and were able to stimulate naïve macrophages as well [13], modulating some inflammatory mediators. Lastly, the infectivity and survival of the *Leishmania* amastigotes within the host cell may also affect the progression of the disease. GP63 is a known important virulence factor, but the role of GP63 in the in vivo modulation of cell recruitment, exosome production, and parasite clearance has not yet been investigated.

In this study, we present a detailed report of the cells responding to infection, the inflammatory environment concerning cytokines/chemokines and exosomes, and the early infection progression in the context of cutaneous leishmaniasis caused by *L*. *major* lacking GP63.

## Materials and methods

### Parasite culture

The wild type *Leishmania* parasite used was NIH S (MHOM/SN/74/Seidman) clone A2. GP63 knockout (GP63^KO^) and GP63 rescue (GP63^R^) *L*. *major* were generously supplied by Dr. Robert McMaster (University of British Columbia, Canada). All parasites were cultured at 25°C, 5% CO2 in Schneider’s Drosophila Medium (SDM) supplemented with 10% heat-inactivated fetal bovine serum (FBS, Wisent, St-Bruno, QC, Canada), and 5mg/ml HEMIN and passaged every 3 to 4 days. Cultures of promastigotes growing at logarithmic phase (day 3–4 post passage) were passaged bi-weekly and were grown to stationary phase (day 6–8 post passage) before being used in infections for all experiments [14].

### Promastigote lysates and western blot analysis

*Leishmania* major cultures grown to promastigote stationary stage were lysed using 7 cycles of freeze-thaw in liquid nitrogen and 42 degrees heating block. Protein levels were dosed with the Bradford Assay (Bio-Rad, Mississauga, ON, Canada). 10% acrylamide gels were loaded with 25 μg of proteins that were added to 5x SDS sample buffer containing bromophenol blue and β-mercapto-ethanol, heated at 95°C for 5 minutes. Electrophoresis was performed at a constant voltage of 100V at room temperature. Proteins were transferred to PVDF membranes (Perkin Elmer, Waltham, MA) using Bio-rad Trans-blot turbo system at 2.5A, 25V for 15 minutes. Membranes were blocked with 5% skim milk for 1 hour and then incubated with the anti-GP63 (Dr. McMaster, University of British Columbia) primary antibody in 5% BSA in TBS-T (TBS- 0.05% Tween 20). It was washed 3 times with TBS-T and incubated with mouse secondary anti-HRP-conjugated antibody (1:10000 in 5% milk) and proteins were visualized by ECL Western Blot Detection System (GE Healthcare, Chicago, IL, USA).

### Gelatin zymography assay

Protease activity of GP63 was assayed using a 10% SDS-PAGE incorporated with gelatin (1mg/ml) as we previously described [15]. Gels were loaded with 5ug of proteins that were added to SDS-PAGE sample buffer (15.6mM Tris pH6.8, 2% SDS, 10% glycerol, 0.05% Bromophenol Blue). Electrophoresis was performed at a constant voltage of 100V, at 4 degrees Celsius. After electrophoresis, SDS was washed with washing buffer (2.5% Triton X-100 in 50mM Tris pH 7.4, 5mM CaCl_2_, 1μM ZnCl_2_) for 1 hr on a shaker at room temperature. The gels were then briefly rinsed twice with deionized water and incubated in a renaturation buffer (50mM Tris pH 7.4, 5mM CaCl_2_, 1μM ZnCl_2)_, overnight at 37°C. After incubation, gels were stained 30 min in 0.5% Coomassie Brilliant Blue R-250 in 30% ethanol and 10% acetic acid and destained by rinsing in a solution containing 30% ethanol and 10% acetic acid until clear bands could be seen. Clear bands on the gel indicated active GP63 activity.

### Mice and ethics

Animal experiments were performed in compliance with the Canadian Council on Animal Care (CCAC) Guidelines, and McGill University Animal Care Committee (UACC). The approved animal use protocol number is 7791. Isoflurane was used for anesthesia prior to euthanasia to alleviate suffering. Mice were housed socially in 3–5 mice per IVC cage, with food, water, and soft bedding.

Mouse experiments were performed in the McGill University Health Centre research institute in containment level 2 housing facilities. Female C57BL/6 adult (6–8 weeks old) mice were used for all experiments, purchased from Charles River Laboratories (Wilmington, MA, USA).

### Footpad infections

Groups of 5 mice were each infected with 5x10^6^ wildtype *Leishmania major* parasites, *L*. *major* GP63 ^KO^ and *L*. *major* GP63 ^R^ injected into one hind footpad. There were 15 mice used in total, 5 per group in 3 groups, with the uninfected footpad being the negative control. No randomization or blinding was used, and mice in each group were housed in the same cage for the duration of the experiment. Lesion development was monitored weekly by the difference of footpad thickness between the infected and uninfected footpad, measured by digital callipers. Cutaneous leishmaniasis progression was monitored over the course of 10 weeks. Mice were euthanized after 10 weeks using isoflurane and CO2 asphyxiation followed by cervical dislocation. There was no humane endpoint and the footpad swelling causes minimal pain and distress.

### Intraperitoneal inoculation

Groups of 3 mice each were infected with 10^8^ wildtype *Leishmania major* parasites, *L*. *major* GP63 knockout, and *L*. *major* GP63 rescue, injected into the intraperitoneal cavity. PBS was used as a negative control. No randomization or blinding was used, and mice in each group were housed in the same cage for the duration of the experiment. There were 12 mice in total, 3 per group in 4 groups. 6 hours post infection, the mice were sacrificed using isoflurane and CO2 asphyxiation followed by cervical dislocation. and 5ml of ice-cold endotoxin-free PBS was used to obtain lavages of the cavities. The number of live cells present in the lavages was counted using a hemocytometer.

Cells were prepared for microscopy using the Cytospin 4 cytocentrifuge (Thermo Scientific, Waltham, MA, USA). The cells were fixed and stained using the Differential Quik (diff-quik) Stain Kit (Ral Diagnostics, Martillac, France). The percentage of cell types found in the lavage was counted. Next, the percent of cells infected and the number of *Leishmania* amastigotes found within the cells were counted.

Two hundred ul of the lavages were plated in 4 well chamber slides and supplemented with Dulbecco’s modified eagle medium (Wisent, St-Bruno, QC, Canada) with 10% FBS and 1% penicillin-streptomycin-glutamine. Cells were kept at 37 degrees Celsius with 5% CO2. 24 hours and 48 hours post plating, cells were fixed and stained using the diff-quik stain kit. The percent of cells infected and the number of *Leishmania* amastigotes found within the cells were counted. From the total 200 cells counted from each slide, the percentage was calculated, and the number of amastigotes found in individual cells was counted as well. These numbers were then used to calculate the total number of amastigotes found within cells by multiplying the percentage infected with the average number of amastigotes per cell.

Total lavage was centrifuged at 1500 rpm for 10 mins to separate cells and supernatant. The cell pellet was resuspended in TRIzol reagent (Ambion life technologies) and frozen.

### Flow cytometry

Mouse peritoneal cell suspensions were stained with the following fluorescence-conjugated mAbs: CD3-BUV737 (17A2) (BD Biosciences, Franklin Lakes, NJ, USA), CD4-FITC (GK1.5) (BD Biosciences), CD8-V500 (53–6.7) (BD Biosciences), CD11b-e450 (M1/70) (Invitrogen), CD11c-PerCP-Cy5.5 (HL3) (BD Biosciences), CD19-APC (1D3) (Invitrogen), CD49b-PE (DX5) (BD Biosciences), F4/80-PE-Cy7 (BM8) (Invitrogen), and Ly6G-Alexa700 (BioLegend, San Diego, CA, USA). Non-viable cells were excluded using fixable viability dye eFluor780 or 506 reagent (Thermo Fisher Scientific). Flow data were collected using a FACS Fortessa X-20 flow cytometer (BD Biosciences), and results were analyzed using FlowJo version 9 software (TreeStar, Ashland, OR, USA).

### Multiplex cytokine/chemokine quantification assay

100ul of the lavage supernatant were analyzed by a multiplex mouse cytokine array/chemokine array 44-plex assay (Eve Technologies, Calgary, AB, Canada). These include Eotaxin, Erythropoietin, 6Ckine, Fractalkine, G-CSF, GM-CSF, IFNB1, IFNγ, IL-1α, IL-1β, IL-2, IL-3, IL-4, IL-5, IL-6, IL-7, IL-9, IL-10, IL-11, IL-12 (p40), IL-12 (p70), IL-13, IL-15, IL-16, IL-17, IL-20, IP-10, KC, LIF, LIX, MCP-1, MCP-5, M-CSF, MDC, MIG, MIP-1α, MIP-1β, MIP-2, MIP-3α, MIP-3B, RANTES, TARC, TIMP-1, TNFα, and VEGF. The multiplex laser bead technology utilizes antibodies that are coupled to colour-coded polystyrene beads where lasers activate the fluorescent dye and excites the fluorescent conjugate, which is then quantified for the concentration of the target analyte. From the data provided from Eve technologies, the total pg of cytokine found in the lavage was calculated from the observed concentration by multiplication of the 5ml PBS used to obtain lavages.

### RNA-seq

Mouse peritoneal cells were recovered, and total RNA was extracted using the EasyPure RNA Extraction Kit (Civic Bioscience) with on-column DNase I gDNA digestion. The RNA integrity was assessed on a Bioanalyzer RNA kit, followed by mammalian rRNA depletion and stranded library preparation using the Kapa Hyperprep RNA kit. The indexes libraries were sequenced on an Illumina NovaSeq 6000 in a paired-end 50bp configuration. The quality of sequence reads obtained for each sequence read was confirmed using the FastQC tool (Babraham Bioinformatics). The reads were trimmed using the paired end mode with the default settings in Trimmomatic [16] and quality was re-assessed using FastQC. Reads were mapped to the mouse UCSC mm9 reference assembly using hisat2 v2.2. [17] and gene expression was quantified by counting the number of uniquely mapped reads with featureCounts using default parameters [18]. TMM normalization and differential expression analysis were conducted using the edgeR Bioconductor package [19]. We retained genes that had an expression level of a minimum of five counts per million reads in at least 3 of the 12 samples, and pairwise differential gene expression analysis was performed by comparing *L*. *major* infected samples versus uninfected samples. Genes with changes in expression ≥|2| and adjusted p-values (<10^−3^) were considered significant. For RNA-seq data visualization in IGV, bigwig files scaled per million reads mapped to exons were generated using genomeCoverageBed and wigToBigWig tools. Relative gene expression heatmaps were created using Cluster 3.0 [20] and visualized in Java Treeview 3.0 [21] Gene ontology analyses were carried out using the online tool Enrichr (Kuleshov et al., 2016) [22], and graphed using GraphPad-PRISM v9.

### Exosome extraction

The remainder of the lavage supernatant was used for the extraction of exosomes. The supernatant was filtered using a 0.22 μm filter to exclude debris and larger vesicles. Lavages from three mice in an experimental group were pooled in 17 mL thin-wall polypropylene tubes (Beckman Coulter, Brea, CA, USA) and were completed with exosome buffer (137mM NaCl, 20mM HEPES). The tubes were centrifuged at 100000xg (RCFavg) for 1 hour at 4°C in an SW 32.1 Ti swinging bucket rotor (Beckman Coulter). The supernatant was discarded, and fresh exosome buffer was added to the tube to wash the pellet. It was centrifuged again at 100000xg for 1 hour. Again, the supernatant was discarded with about 400ul liquid remaining and the exosome pellet was resuspended in the remaining exosome buffer and frozen for further analysis. An alternate method used to extract exosomes included the ExoQuick procedure as described in the protocol for ExoQuick exosome precipitation solution (System Biosciences, Palo Alto, CA). This step was performed in place of the second ultracentrifugation step mentioned above. The protein levels were dosed using a microBCA assay (Thermo Fisher, Waltham, MA, USA).

### Nanoparticle tracking analysis

The exosomes were then analyzed using nanoparticle tracking analysis (NTA) using the NanoSight NS500 (Malvern Panalytical, Malvern, Worcestershire, UK) in the laboratory of Dr. Janusz Rak. Samples were diluted with exosome buffer and injected into the sample chamber. 3 videos were captured for 30 seconds each at 37 degrees Celcius, using optimized camera settings that were kept consistent for all samples. From the NTA analysis, the concentration and mean, median, mode size of the particles of all particles were calculated and graphed [23]. After the size and concentration of the particles were verified for proper exosome isolation prep, transmission electron microscopy photos were taken to further confirm their isolation and purity.

### Transmission electron microscopy

EVs were suspended in exosome buffer. Samples were deposited onto Fomvar carbon grids (Mecalab, Montreal, QC, Canada), fixed with 1% glutaraldehyde in 0.1M sodium cacodylate buffer, and washed 3 times with autoclaved Milli-Q, and stained with 1% uranyl acetate. Each aforementioned step was performed for 1 minute in duration. The FEI Technai-12 120kV transmission electron microscope and AMT XR80C CCD Camera (Facility for Electron Microscopy Research, McGill University, Montreal, Canada) were used to visualize samples.

### Trichloroacetic acid (TCA) precipitation

Exosome solution with 8ug of protein was aliquoted and completed up to 100ul with ddH2O. To the exosomes, the following were added: 100ul 10X TrisHCL-EDTA, 100ul 0.3% sodium deoxycholate, 72% TCA. The tubes were incubated on ice for 1 hour, then spun at 14000 rpm for 20 minutes at 4 degrees. The supernatant was aspirated and the pellet was resuspended in 100ul of 90% room temperature acetone. The tubes were then incubated in the -20°C freezer overnight, then centrifuged at 14000 rpm for 20 minutes at 4°C. The supernatant was aspirated then the pellet was air dried at room temperature and placed at -20°C.

### Liquid chromatography-mass spectrometry (LC-MS/MS)

Liquid chromatography tandem mass spectrometry (LC-MS/MS) was performed at the Institut de Recherches Clinique de Montreal (Universite de Montreal, Montreal, QC, Canada). 8ug of proteins from extracted EVs were precipitated with 15% trichloroacetic acid/acetone and sent for LC-MS/MS analysis. After precipitation, proteins were reduced, alkylated, and digested with trypsin solution (5mg/ul trypsin sequencing grade from Promega, 50mM ammonium bicarbonate). Protein digestion was performed at 37 degrees for 18h and stopped with 5ul of 5% formic acid. Prior to LC-MS/MS, digests were cleaned using C18 ZipTip pipette tips (Millipore, Burlington, MA, USA). Extracted peptides were injected into a Self-Pack PicoFrit fused silica capillary column (New Objective) of 15 cm long and packed with the C18 Jupiter 5 μm 300 Å reverse-phase material (Phenomenex) and chromatographically separated on an Easy-nLC II system (Proxeon). Eluted peptides were electrosprayed from a Proxeon nanoelectrospray ion source and analyzed on a LTQ Orbitrap Velos mass spectrometer (ThermoFisher Scientific).

### Protein database search

The peak list files were generated with Proteome Discoverer (version 2.1) using the following parameters: minimum mass set to 500 Da, maximum mass set to 6000 Da, no grouping of MS/MS spectra, precursor charge set to auto, and the minimum number of fragment ions set to 5. Protein database searching was performed with Mascot 2.6 (Matrix Science, Boston, MA, USA) against the RefSeq and Uniprot *Mus Musculus* protein database. The mass tolerances for precursor and fragment ions were set to 10 ppm and 0.02 Da, respectively. Trypsin was used as the enzyme allowing for up to 1 missed cleavage. Cysteine carbamidomethylation was specified as a fixed modification and methionine oxidation as variable modifications. MS/MS peptide and protein identifications were performed using Scaffold software version 4.8.9 (Proteome Software Inc., Portland, OR, USA). Peptide identifications were included if they could be established at greater than 95.0% probability by the Peptide Prophet algorithm with Scaffold delta-mass correction. Protein identifications were accepted if they could be established at greater than 80.0% probability and contained at least 2 identified peptides in at least one biological replicate [24] The proteins sharing significant associated probability were grouped into clusters.

### Bioinformatics analysis

Normalization, quantification, and comparisons of proteins from lavage EVs were performed using the Scaffold software. Visualizations of set intersections in a matrix layout were generated by UpSetR [25]. Gene Ontology comparisons were performed using Panther (www.pantherdb.org) [26].

### Statistical analysis

Statistical analysis was performed in Graphpad Prism 6.0c (La Jolla California USA).

## Results

### GP63 protein and proteolytic activity are absent from *L*. *major*^KO^

**Fig 1:**
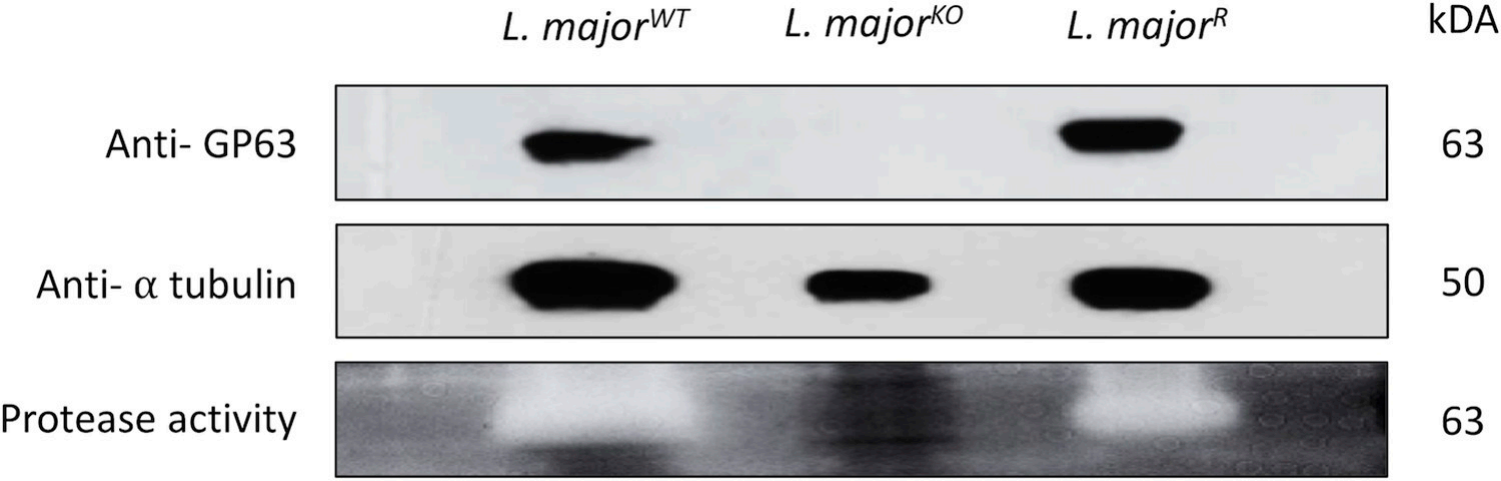
Validation of active GP63 in *L*. *major* lysates. Protein extracts from *L*. *major*^WT^, *L*. *major*^KO^, and *L*. *major*^R^ were loaded on gel to confirm GP63 expression and proteolytic activity. Top two rows indicate western blot (WB) results. Bands were seen at 63kD for GP63 in WT and GP63 rescue parasites only, bands at 50kD for alpha-tubulin were seen for all *L*. *major* lysates. The bottom is a gel doc image for the activity of GP63 found at 63kD in WT and GP63 rescue *L*. *major*.

Prior to experimentation with existing *Leishmania*, it was essential to verify the stable deletion and expression of GP63 in our transgenic cultures [9]. Western blot analysis was used to detect the presence of GP63 using a mouse anti-GP63 antibody. The assay detected a band corresponding to GP63 at 63kD in the lysates of *L*. *major*^WT^ parasite as well as *L*. *major*^R^ (Fig 1). No bands were seen in the *L*. *major*^KO^ lane. Gene expression of GP63 was also verified using RNAseq to account for any genetic changes through prolonged lab culturing (S2A Fig). The transcriptome profile demonstrated our *L*. *major*^KO^ did not express any of the GP63 genes. Gelatin zymography assays are useful for the detection of active proteases, which will degrade the gelatin copolymerized with the SDS gels [27]. The gelatin assay demonstrated there was no metalloprotease activity found in the protein lysate of the GP63 knockout *L*. *major*. Clear bands were only observed in the lanes containing *L*. *major*^WT^ and *L*. *major*^R^, indicating the presence of active GP63 [28].

### *Leishmania major* lacking GP63 generates a less aggressive lesion development

**Fig 2:**
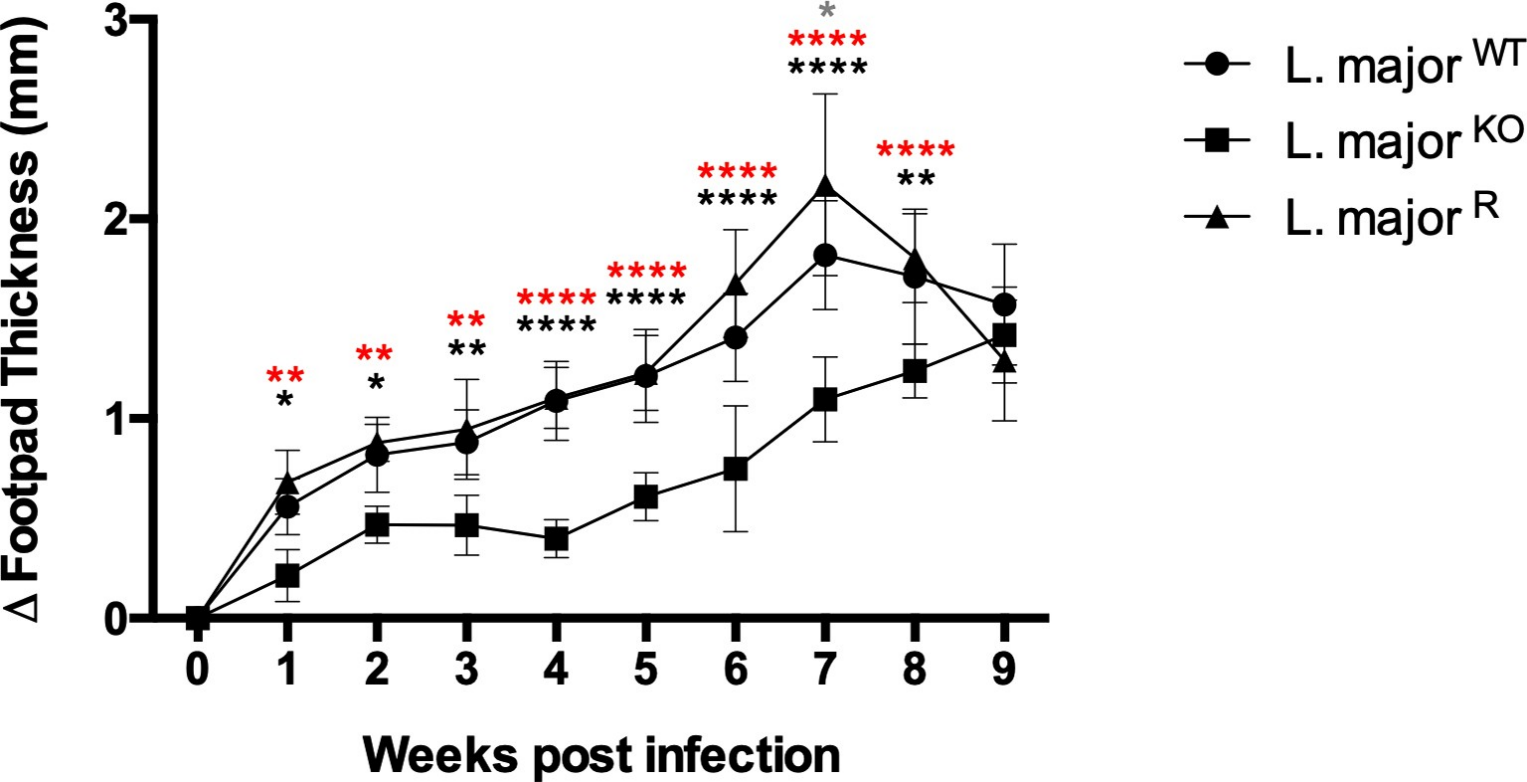
Footpad lesion formation progression by *L*. *major* inoculation is GP63 dependent. Graph tracking the difference in thickness between the inoculated and the uninfected footpad over ten weeks. Bars represent ± SEM (n = 5 mice per group). Statistical significance was determined using two-way ANOVA between 3 groups, *L*. *major*^WT^ and *L*. *major*^KO^ (black *), *L*. *major*^KO^ and *L*. *major*^R^ (red *), *L*. *major*^WT,^ and *L*. *major*^R^ (grey *). Where *p<0.05, **p<0.01, ***p<0.001, ****p<0.0001. Each time point was analyzed individually, without assuming a consistent SD. No data points or mice were excluded.

The progression of lesion formation in the footpad infection was monitored over ten weeks. It was previously described that infection with GP63 deficient *Leishmania* promastigotes resulted in the delay of lesion formation in the footpad of BALB/c mice, a strain susceptible to infection [8, 9, 29]. We infected three groups of 5 *L*. *major* resistant C57BL/6 mice with 1 x 10^6^ promastigotes to confirm this observation in our mouse model. Over the first 9 weeks, the initial difference of footpad thickness between the infected and uninfected feet in *L*. *major*^KO^ infected mice was significantly lower compared to the wild type parasites, at about 0.5mm less (Fig 2) (p<0.05, multiple T-tests Holm-sidak method) in the first 2 weeks post-infection. As the infection progressed, the footpad thickness difference in *L*. *major*^KO^ parasites started to increase up to 1mm after week 5, demonstrating a delay in the lesion formation. The *L*. *major*^WT^ and *L*. *major*^R^ parasites induced a more significant swelling progressively as the infection established over eight weeks, peaking at 2mm difference in thickness, which subsequently began to subside by week 8. By week 9, *L*. *major*^KO^ infected footpads reached 1.5mm, compared to *L*. *major*^WT^ and *L*. *major*^R^ groups, which were on their decline. Data stemming from this experiment have confirmed that the loss of GP63 weakens the ability to produce progressive lesions when compared to wild type and rescue parasites.

### *L*. *major*-induced inflammatory cell recruitment is not influenced by GP63

An intraperitoneal injection model was used to characterize the early innate immune response to an *L*. *major* infection. The mouse peritoneal cavity is ideal for the study of the innate inflammatory response, as it contains mainly macrophages, followed by neutrophils and to a lesser extent, eosinophils, basophils, B cells, T cells and is permissive to the recruitment of a variety of immune cells [30]. In addition, it was observed that antigenic stimulation in the intraperitoneal cavity results in the recruitment and activation of inflammatory cells [31]. It is difficult to study the inflammatory milieu such as cytokines and exosomes in the cutaneous models of infection such as footpad or ear injection. The peritoneal cavity is the ideal in vivo environment for an in-depth study of innate immune system modulation. From the peritoneal lavage, we sought to study the number and type of cells recruited, the production of inflammatory cytokines and chemokines, and exosomes released.

**Fig 3:**
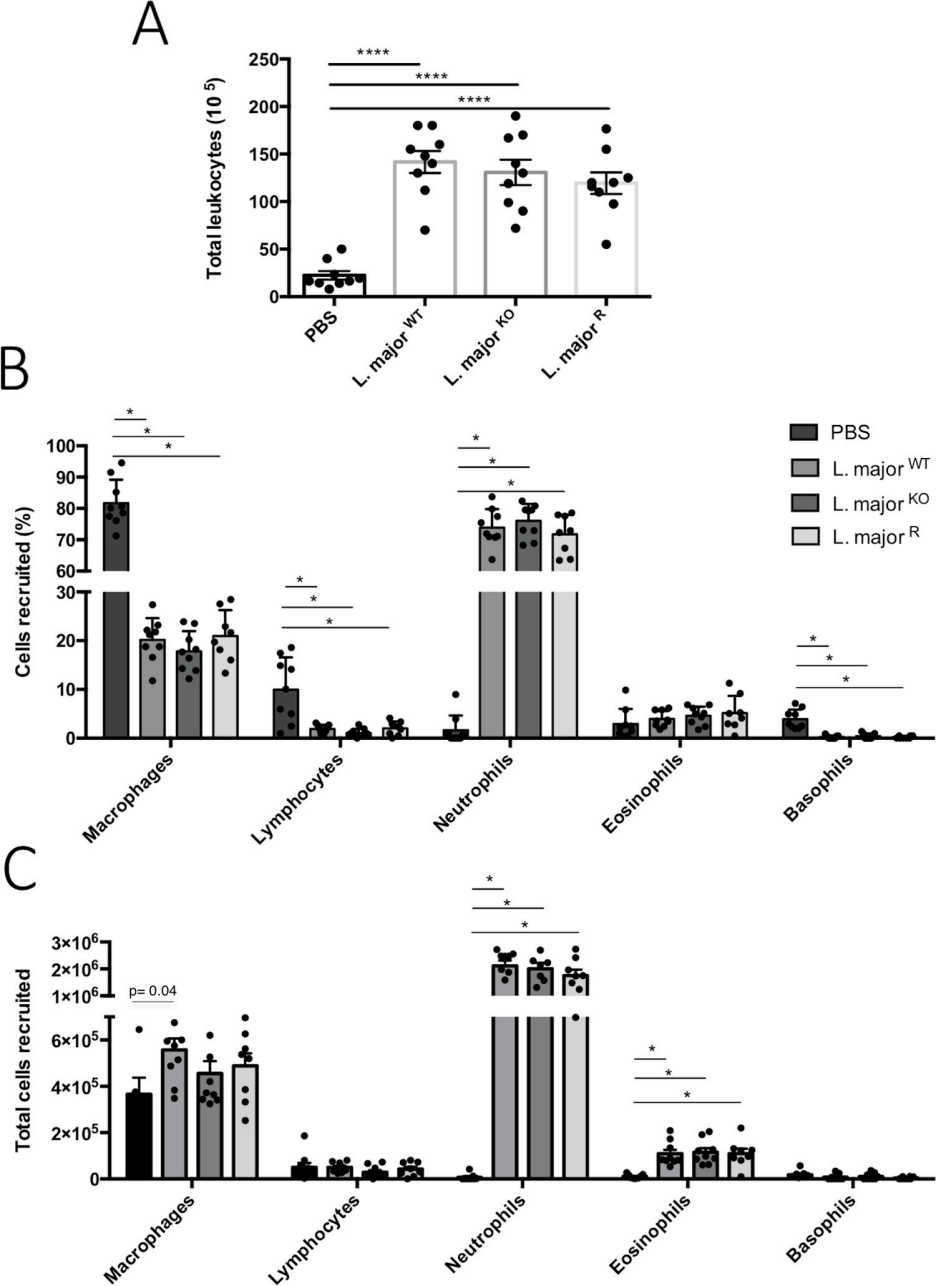
GP63 does not affect inflammatory cell recruited in response to *Leishmania* inoculation. A) Total live cells retrieved in intraperitoneal lavage 6-hr post-inoculation of infection with *L*. *major*^WT^, *L*. *major*^KO^, and *L*. *major*^R^. Cells were counted using a hemacytometer; values were calculated based on concentration and total lavage volumes. Bars represent ± SEM (n = 9, mean of 3 independent experiments). Statistical significance was determined using unpaired T-tests with Welch’s correction, ****p < 0.0001. Endotoxin-free PBS was used as inoculation control. B) Distribution of inflammatory cell types as a percentage of the total. Statistical significance was determined using Multiple T-tests with Holm-Sidak method, with alpha = 5.000%, *p<0.05. C) The total amount of each cell type was calculated using the total live cells in A and the percentage of each B. The same statistical test was applied in B. No data points were excluded.

The number of live cells was counted from the intraperitoneal lavage taken 6 hours post-infection. In the PBS injected control group, the total amount of live cells in the lavage was 2.2x10^6^ cells (Fig 3A). In the infection groups of *L*. *major*^WT^, *L*. *major*^KO^, and *L*. *major*^R^, the average total amounts were 1.4x10^7^, 1.3x10^7^, and 1.2x10^7^ cells, respectively. There was no significant difference between the three infection groups, regardless of the presence of GP63. On the other hand, *L*. *major* injection resulted in the five-fold increase of cells in the peritoneal cavity (p<0.0001, unpaired t-tests with Welch’s correction) in comparison to PBS inoculated mice.

The inflammatory cell types recruited have been monitored from Diff-Quick cytospin slides. In the PBS injected control group, 80% of the cells retrieved were macrophages or monocytes. The remaining cells consisted of 10% lymphocytes, and small amounts of neutrophils, basophils, and eosinophils (Fig 3B). Compared to the PBS group, all *Leishmania* infections resulted in massive recruitment of leukocytes, mostly neutrophils (p<0.05, multiple t-tests Holm-sidak method). The total amount of neutrophils found in the lavage was about 2x10^6^ cells in all three infection groups, compared to the 6x10^3^ in the PBS lavage, which is a ~300 fold difference (Fig 3C). There was also a significant increase in eosinophils for all 3 *L*. *major* infection groups. The eosinophil counts increased tenfold from 1x10^5^ cells to around 1x10^6^ cells for all three groups. Macrophage counts also increased slightly in the *L*. *major* WT infection group, (p<0.05, multiple t-tests Holm-Sidak method), while the total number of lymphocytes and basophils remained in a similar range. The most notable observation is that all three groups of *L*. *major* infected mice had similar amounts of each type of recruited cells, regardless of GP63. The cell recruitment levels were similar for all three groups for all cell types, which consisted mainly of neutrophils. GP63 did not affect the number of cells recruited to the site of infection, nor the type of cells that were recruited.

**Fig 4:**
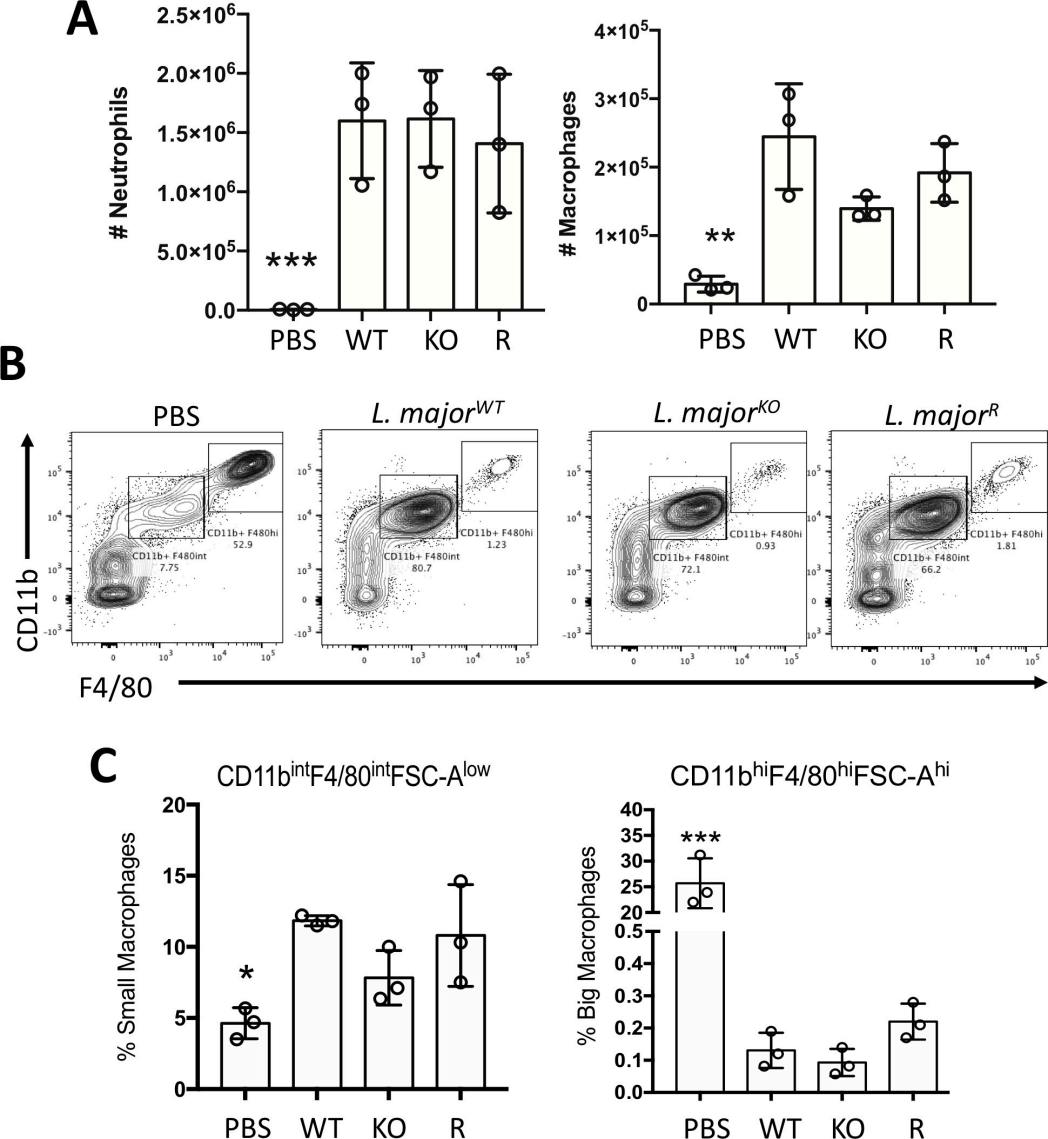
Flow cytometry data of cell recruitment and polarization of two distinct macrophage populations observed in mice intraperitoneal lavage following a 6-hour infection. A) Total numbers of neutrophils (CD11c+, Ly6G+, lymphoid-) and macrophages (lymphoid-, CD11c-, F4/80+, CD11b+), were calculated using the flow cytometry data and the total number of cells. Bars represent ± SD, and each point represents one mouse, n = 3 mice per group, ****p<0.0001. B) Flow cytometry gating of small and large peritoneal macrophages (lymphoid-, CD11c-, F4/80+, CD11b+) C) Percentage of small and large peritoneal macrophages. Bars represent ± SD, and each point represents one mouse, n = 3 mice per group, *p<0.05, ***p<0.001.

Flow cytometry was also used to quantify the level of cell recruitment and further elucidate the identity of the cells found in the intraperitoneal lavage (Fig 4A). Cytometry data corroborated with the neutrophil and macrophage cell count numbers observed in the cytospin slides (S1A Fig).

The macrophages present in the peritoneal cavity were further analyzed to distinguish the two functionally distinct macrophage subsets that can vary due to antigenic stimulation [32]. Large peritoneal macrophages make up 90% of peritoneal macrophages in unstimulated conditions, and small macrophages become dominant after stimulation of LPS, hypothesized to derive from active monocytes migrating to the peritoneum upon inflammation (S1B Fig). The flow cytometry data was further analyzed to identify these distinct populations, where cells were gated for macrophage surface markers CD11b and F4/80 (Fig 4B). All three infection groups demonstrated a shift in macrophage populations from mostly large peritoneal macrophages (LPM; lymphoid-, CD11c-, F4/80+, CD11b+) to small peritoneal macrophages (SPM; lymphoid-, CD11c-, F4/80lo, CD11b+). Six hours post-infection, the percentage of small macrophages increased over two-fold while the large macrophage population diminished significantly (Fig 4C). The difference between wild type, GP63 rescue, and GP63 knockout infected groups was not significant.

Collectively, this set of data reveals that the *Leishmania* parasite can rapidly induce the recruitment of inflammatory cells at the site of inoculation, but that does not require the metalloprotease GP63. Therefore, this inflammatory event occurring during *Leishmania* infection cannot provide clues why *L*. *major*^KO^ concur to less aggressive skin pathology.

### Inflammatory mediators released in response to *Leishmania* intraperitoneal infection

**Fig 5:**
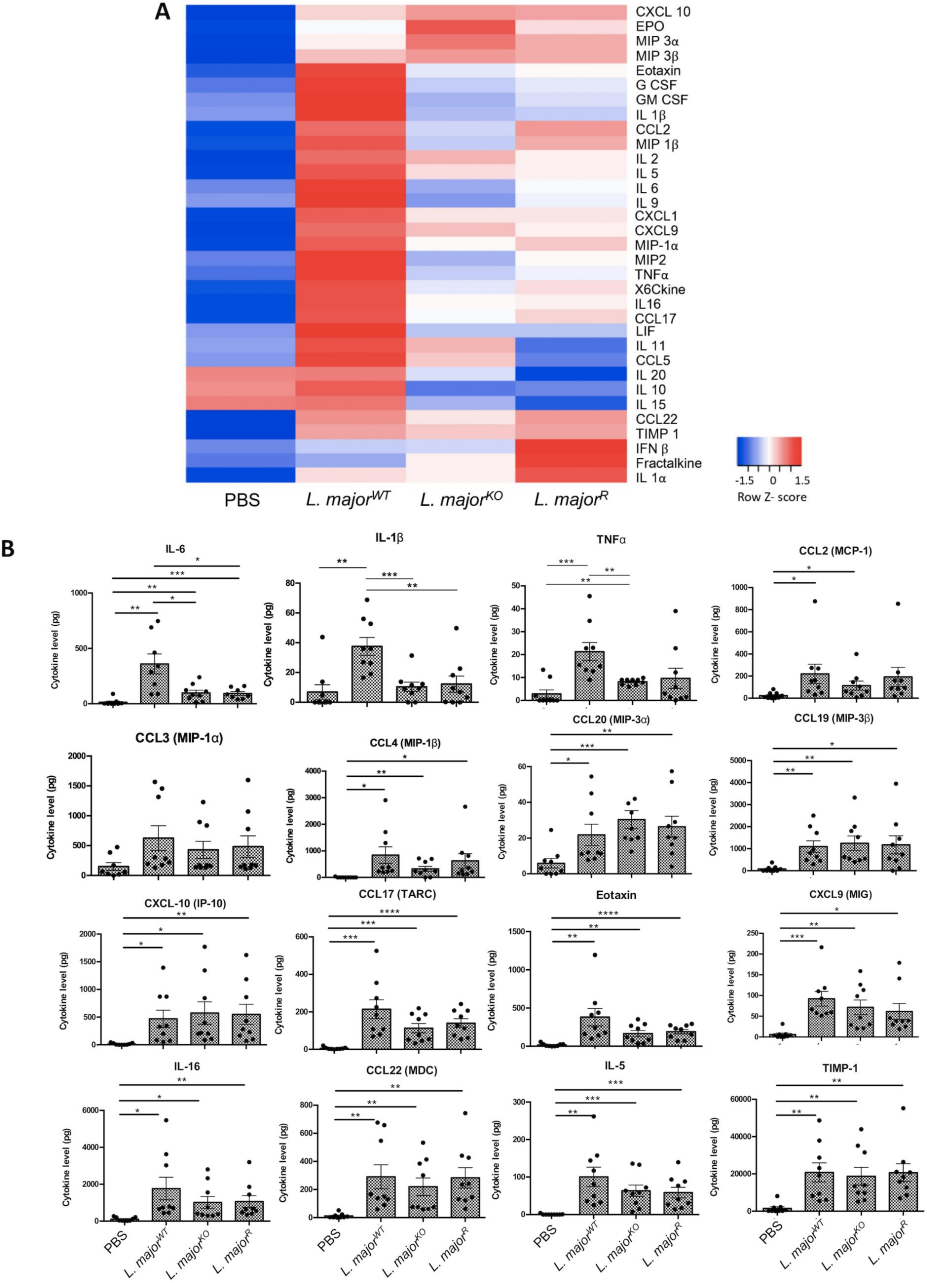
Proinflammatory mediators and chemokine expression are modulated by *L*. *major* infection. A) Expression heat map generated from the multiplex assay analyte data. The analysis was performed on total supernatant retrieved from intraperitoneal lavages following a 6-hour infection with 10^8^ WT, GP63^KO^, and GP63^R^ *L*. *major*. Each row is analyzed individually for relative expression levels. B) Cytokine levels in pg measured from the multiplex assay. Total cytokine was calculated using the volume of lavage and concentration, as reported by fluorescence readout. Bars represent ± SEM, each point represents one mouse, n = 9 (mice total, 3 mice per experiment) Statistical significance was determined using unpaired t-tests with Welch’s correction, *p<0.05, **p<0.01, ***p<0.001, ****p<0.0001.

We sought to observe the cytokines and chemokines produced during infection, which will describe the inflammatory environment, as well as explain the observed recruitment of cells. The peritoneal lavage supernatants were directly measured using a multiplex cytokine/chemokine array to quantify protein levels. From the average of all nine mice from 3 experiments, some general trends were observed. The lowest concentration was observed for most cytokines in mice injected with PBS (Fig 5A). In contrast, the mice infected with *L*. *major*^WT^ produced the highest levels of cytokines. High cytokine level was not observed in the group inoculated with *L*. *major*^R^, despite expressing similar levels of active GP63. The *L*. *major*^KO^ inoculated mice also showed similar production of cytokines/chemokines to *L*. *major*^R^, which overall correlates well with the inflammatory cell recruitment data. The *L*. *major*^WT^ infected mice produced the highest levels of proinflammatory cytokines when compared to both *L*. *major*^KO^ and *L*. *major*^R^: IL-6 (~4 fold, p<0.05), IL1- β (~4 fold, p<0.001), and TNF-α (~3 fold, p<0.01) (Fig 5B). The elevated pro-inflammatory mediator levels observed solely in the *L*. *major*^WT^ were not reflected in the inflammatory cell recruitment numbers. Further immunological events beyond the innate inflammatory events could have influenced the infection, but from *L*. *major*^R^ data does not correlate with the difference of infection level seen between *L*. *major*^KO^ and *L*. *major*^R^. Statistically, there was no significance between all groups for the chemokines and other cytokines. These results corroborate with those seen in the cell recruitment numbers, which were similar between all three infection groups, with mostly neutrophils observed. From this information, GP63 did not affect the immune mediators produced from the peritoneal cells.

### Transcriptional profiles of peritoneal cells during *Leishmania* infection

**Fig 6:**
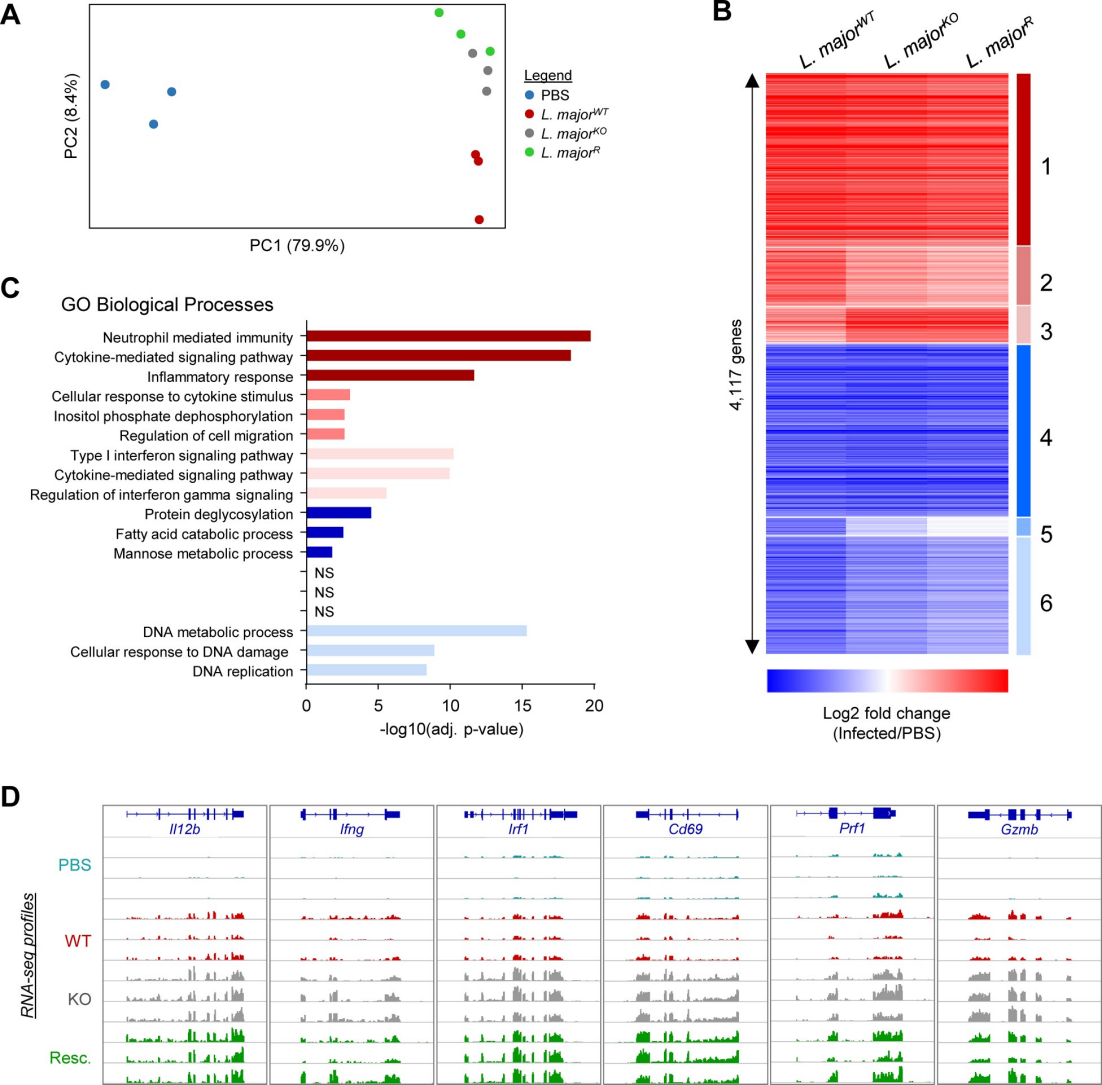
Transcriptional changes of peritoneal cells during *Leishmania* infection. RNA sequencing of peritoneal cells at 6 hours following control PBS injection or *L*. *major* (WT, KO, or Rescue) inoculation. A) Dimension reduction analysis (partial least-squares) of the RNA-seq samples. B) Heatmap showing K-mean clustering of the genes differentially expressed between the non-infected (PBS) and at least one of the infected conditions; genes with fold change ≥ |2| and adjusted p-value ≤ 0.001 were considered significant. Complete analysis C) Gene ontology enrichment analysis showing the top 3 enriched categories for each of the 6 clusters of differentially expressed gene clusters shown in B (complete enrichment data available in S2 Table). D) Genome snapshot showing example of genes from cluster #3 demonstrating a stronger interferon response and Th1 and cytotoxic response in *L*. *major* KO (and GP63 rescue)-infected mice (more examples are provided in S2 Fig).

We then analyzed the transcriptome of the peritoneal cells before and following *Leishmania* infection to better understand the changes in cellular proportions and transcription. Six hours post-inoculation with PBS, *L*. *major*^WT^, *L*. *major*^KO^, or *L*. *major*^R^ peritoneal cells were recovered and the ribosomal RNA-depleted total RNA was sequenced (RNA-seq). A dimension reduction analysis highlights the major transcriptional changes induced by *Leishmania* infection (PC1) whereas *L*. *major* KO and GP63 rescue infected samples separate from the WT-infected along with the second principal component (Fig 6A). The KO and rescue of GP63 for the inoculated *L*. *major* were confirmed using the *Leishmania* RNA captured in the peritoneal cell RNA-seq (S2A Fig). A differential gene expression revealed 4,117 genes with significant expression changes (fold change ≥ |2| and adjusted p-value ≤ 0.001) following infection (Fig 6B, S1 Table). Interestingly, while 502 of these genes had a significantly different response for the mutant *L*. *major* compared to the wild-type infected samples, none were different between *L*. *major*^KO^ and *L*. *major*^R^ (S1 Table).

K-mean clustering of the differentially expressed genes highlighted 3 groups of upregulated genes and 3 of downregulated genes, most being similarly affected by the three *L*. *major* (clusters 1, 4 & 6). Given that the sequenced RNA was extracted from all cells found in the peritoneal cavity, our dataset can reveal changes in cellular composition and/or activation status. Hence, we have performed a gene ontology enrichment analysis to explore the biological functions found in each gene cluster (Fig 6C, S2 Table). Confirming the observations made above, infection with all three *L*. *major* induces (cluster 1) neutrophil recruitment, myeloid activation, and production of cytokines/chemokines (e.g. *Trem1*, *Trem3*, *C5ar1*, *Ltf*, *Ptgs2*, *Ccl2*, *Ccl12*, *Ccl17*, *etc*.). On the other hand, genes with a reduction in expression by all three *L*. *major* (clusters 4 & 6) are associated with cell cycle, DNA damage response, oxidation-reduction, nucleosome assembly, and fatty acid metabolic process. Interestingly, despite not being significantly enriched in a GO category because of the limited gene number, cluster #5 includes multiple cellular markers of B cells (e.g. *Cd19*, *Cd79a*, etc.) and mast cells (e.g. *Gata2*, *Cma1*, etc.) (S2B Fig), indicating that these cell types are proportionally less abundant following *L*. *major* ^WT^ infection only. Conversely, cluster #2 contains genes more strongly activated by *L*. *major*^WT^ and are associated with regulation of migration, in particular some chemokine receptors expressed by neutrophils and macrophages (e.g. *Cxcr1*, *Cxcr2*, *Ccr1*). Of particular interest, cluster #3 genes have greater activation in *L*. *major*^KO^ and *L*. *major*^R^ infected conditions; with functions in cytotoxic lymphocytes (e.g. *Prf1*, *Gzma*, *Gzmb*, etc.), the interferon response pathway (e.g. *Ifit2*, *Cxcl9*, *Cxcl10*, *Fos*, *Mx1*, etc.), and the Th1 response (e.g. *Tbx21*, *Irf1*, *Il12b*, *IFNg*, etc.) (Fig 6B–6D, S2C Fig).

### *Leishmania* infection augments exosomes released by host inflammatory cells

Extracellular vesicles are secreted by all eukaryotic cells and it has been shown that infectious stimulation alters exosome release from host cells [33]. Exosomes derived from macrophages infected with *Leishmania* in vitro have been studied, and they were found to have altered effector functions [13]. We extracted exosomes from the total collected supernatant, which represent exosomes secreted from all cells present in the intraperitoneal lavage.

**Fig 7:**
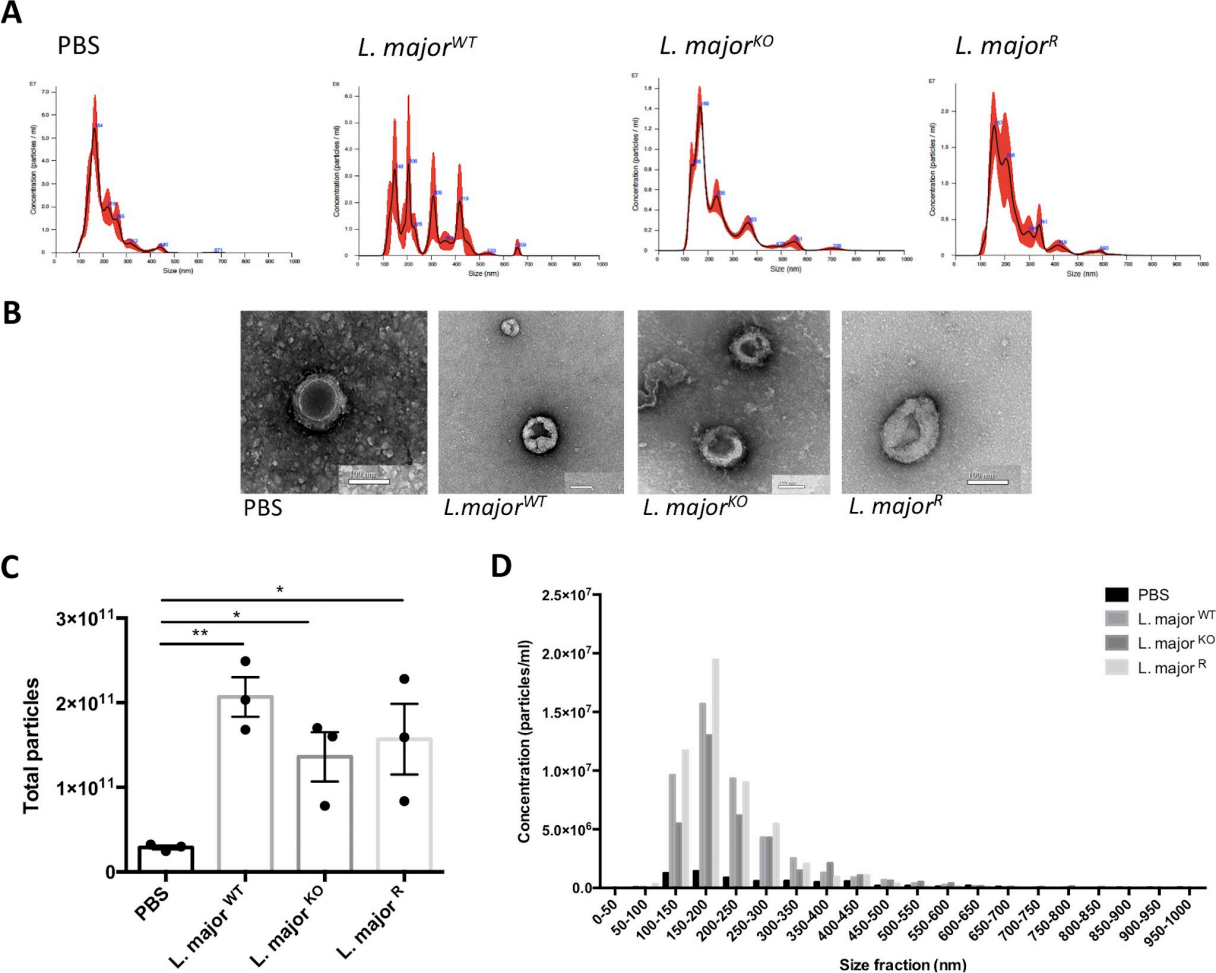
Nanoparticle tracking analysis and transmission electron microscopy for exosome analysis. The analysis was performed on EV suspension extracted from total supernatant retrieved from intraperitoneal lavages following a 6-hour infection with *L*. *major*^WT^, *L*. *major*^KO,^ and *L*. *major*^R^. A) Representative Nanosight generated graphs of nanoparticle distribution and concentration in samples. Average concentration, as calculated in the three videos analyzed. B) TEM photos of exosomes extracted after staining with uranyl acetate to visualize morphology. Photos were taken at 32000x; the scale bar represents 100nm. C) The concentration of nanoparticles found in suspension was quantified used nanoparticle tracking analysis. The total number of particles was calculated using the concentration and total lavage volume. Bars represent ± SEM; each point represents 3 mice pooled into one sample, n = 3 (9 mice total, 3 per experiment). Statistical significance was determined using unpaired t-tests with Welch’s correction, *p<0.05, **p<0.01. D) The concentration of particles found in each size fraction of 50nm. Bars represent the average in one experiment only, n = 3, 3 mice total per group.

Nanoparticle tracking analysis (NTA) was performed, and transmission electron microscopy (TEM) photos were taken to verify the purity of exosomes. NTA analysis showed clear peaks of nanoparticles at about 150nm in size (Fig 7A). The PBS sample contained a lower concentration of exosomes, which cause higher background reading. Isolated exosomes were further visualized using transmission electron microscopy. TEM photos confirmed that the isolation was successful and yielded exosomes of the expected size range and morphology (Fig 7B). The exosome lipid bilayer can be observed in the 30000x photos. Exosomes are about 100nm in diameter and have a round, cup-shaped morphology when fixed onto grids. Most particles seen in the TEM photos were uniform in shape and size.

From the NTA graphs, the mean and mode of the size of the nanoparticles were obtained. The mode extracellular vesicle (EV) size was consistent for all four groups, in the range of about 150nm in diameter. The larger average size for exosomes is due to the overestimation of the Nanosight machine, a common occurrence. The concentration of the total nanoparticles was also measured, along with the concentration of particles in each size fraction. The EV concentrations showed that the PBS groups had ten-fold fewer EVs than the infection groups, which is reflected in the total cell counts (Fig 7C). Wild type and GP63 rescue *L*. *major* infected cells appeared to produce slightly more EVs than the cells infected with GP63 knockout parasites, however statistically insignificant. From the size fraction breakdown, GP63 rescue parasites induced more exosomes than GP63 knockout parasites, as seen in the 100–150 fraction (~2 fold) (Fig 7D). Since the cell counts were similar between the three infection groups, the relative level of exosome production could be attributed to the effects on individual cells themselves.

### Proteomic analysis of EV protein cargo

Exosomes carry cargo that has been shown to have various functions in cell-cell communication, notably immune cell activation and suppression [34]. To further elucidate the immune activation environment during a *L*. *major* infection and the impact of GP63, we performed mass spectrometry (MS) on the total proteins found in the extracted EVs. When MS data was analyzed against the UniProt *Mus musculus* database, a total of 1226 protein hits in 1131 clusters were identified, with at least 2 total spectrum counts in at least one sample. We first identified known exosome markers to verify that our protein dataset reliably represents exosomal proteins (S3 Table). In our samples, CD9, heat shock cognate 71kD protein, glyceraldehyde-3-phosphate dehydrogenase, actin cytoplasmic 1, and annexin A2 were identified in all samples [35]. CD63, another exosome marker, was not found in all samples (S3 Table). The levels for all markers were found to be higher in GP63 knockout and GP63 rescue samples, which may reflect the relative levels of total exosomal protein and need to be accounted for when looking at protein enrichment. There are also signs of protein contamination from non-exosome biofluid sources, for example, serum albumin and keratin, which could also skew data. To calculate the enrichment of proteins, exponentially modified protein abundance index (emPAI) values were used, which is proportional to protein content in a protein mixture [36]. These values were plotted using log10 values to visualize the general pattern of protein up or down regulation following infection (S3 Fig). emPAI values could also be compared using T-test, where -log10 p-values were graphed using a volcano plot (S4 Fig). The number of proteins upregulated between two groups was visualized, and individual proteins could also be identified. Several proteins of interest were expressed more in the *L*. *major*^WT^, *L*. *major*^KO^, and *L*. *major*^R^ infected groups compared to the PBS group. The proteins presented (S3 Table) are known to be involved in immune responses or involved with the activity of immune cells. According to gene ontology terms found on Uniprot, BPI fold-containing family A member 2 and Protein S100-A9 are secreted antimicrobials, Neutrophilic granule protein is a protease inhibitor involved in defence, CD177 antigen, Pentraxin-related protein PTX3, and Myeloperoxidase are involved in neutrophil function, and eosinophil peroxidase is involved in defence in eosinophils.

The number of shared and unique proteins was also analyzed from the total spectrum count. This is presented using an UpSet plot, which is used to visualize intersections of protein sets found within the EVs [25]. Many of the proteins were shared between all 4 groups (337), while the next largest intersection was between GP63 knockout and GP63 rescue groups (248), likely due to the abundance of proteins found in these groups (S5 Fig). Unique proteins could also be identified, where *L*. *major*^KO^, *L*. *major*^R^, *L*. *major*^WT,^ and PBS groups expressed 105, 70, 30, and 10 unique proteins, respectively. From this, we see that infection by *L*. *major* in the absence of GP63 produces a different host response environment compared to infection with wild type *L*. *major*.

The proteins found within the extracellular vesicles could also be categorized based on biological function, molecular function, cellular component, pathway, and protein class through gene ontology. Histograms of the percentage of proteins found in each category were generated using GO terms in Scaffold. The percentage of proteins categorized into different biological process groups and molecular function groups were found to be similar between all 4 experimental groups. (S6 Fig). As observed in cell recruitment, cytokine production, and exosome proteome, the host inflammatory response does not seem to be affected by the absence or presence of GP63.

### *Leishmania* GP63 favour parasite survival and growth within macrophages

From analyzing our cell recruitment, cytokine production, and exosome data, we were unable to conclude as to why *L*. *major*^KO^ footpad infection led to a reduced and delayed lesion formation. Therefore, we moved on to analyze the infectivity and survival of parasites over time. A common way that leishmaniasis is diagnosed clinically is to observe stained cells obtained in lymph nodes for amastigotes within monocytes and macrophages [37]. From the cytospin prepared Diff Quik stained slides, amastigotes could be seen as round or oval bodies within the cytoplasm of infected cells. Amastigotes were observed in cells from all 3 *L*. *major* infected groups, in both neutrophils and macrophages.

**Fig 8:**
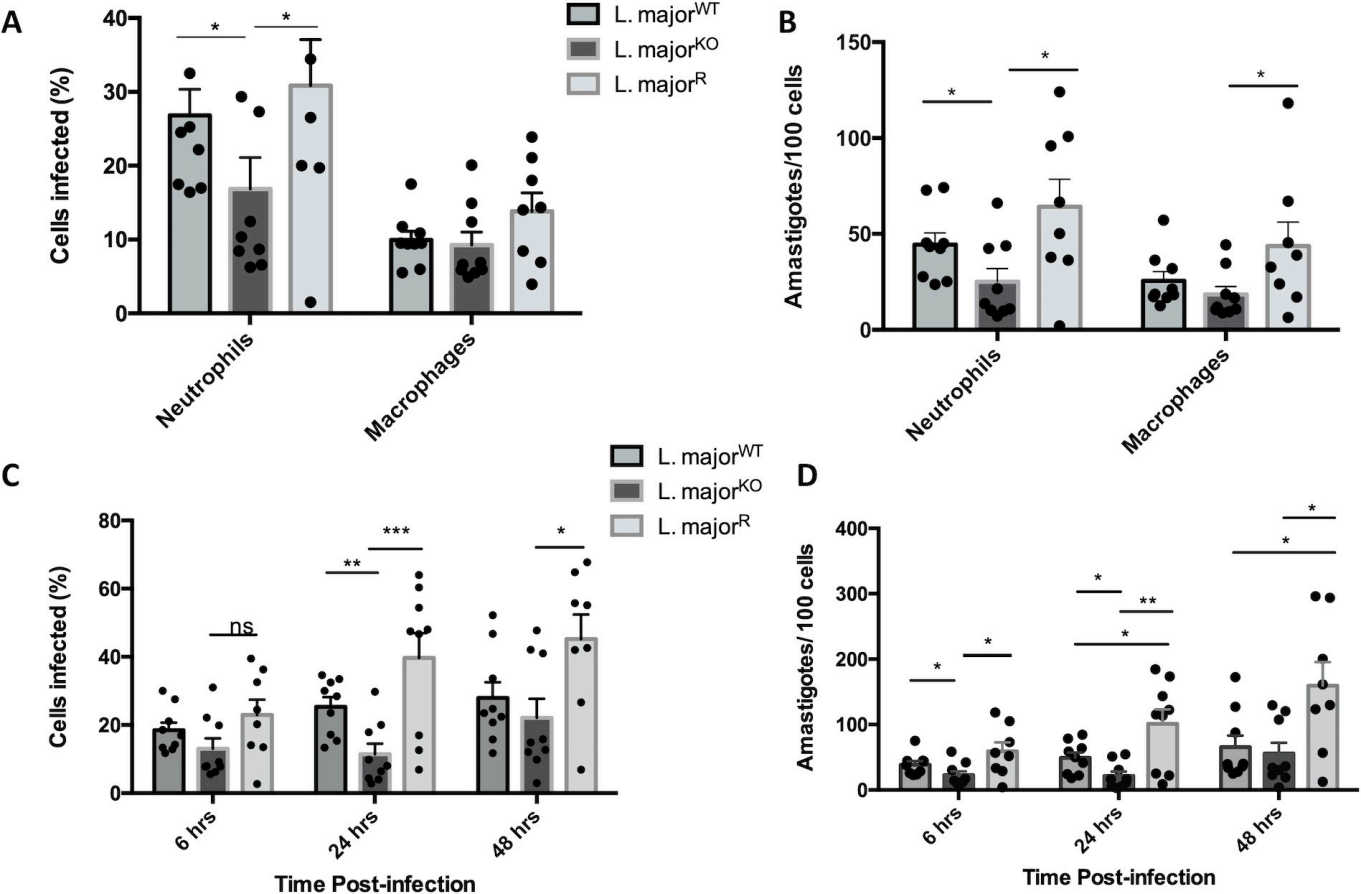
GP63 increases *Leishmania* infection and number of amastigotes in cells over time. A) Percentage of neutrophils and macrophages infected. 200 cells were counted, bars represent ± SEM, each point represents one mouse, (n = 9 mice total, 3 mice per experiment) Statistical significance was determined using unpaired t-tests with Welch’s correction, *p<0.05. B) The number of amastigotes found per 100 cells, which was determined using the average amastigotes found per cell and the percentage of infected cells. The same statistical test was applied as A). Intraperitoneal lavages following a 6-hour infection with *L*. *major*^WT^, *L*. *major*^KO^, and *L*. *major*^R^ were plated onto chamber slides. C) Percentage of cells infected at different time points post-infection. Data for 6 hrs was from cytospin prepared slides, and cells were fixed and stained at 24hrs and 48hrs post-infection. The same statistical test was applied as in A). D) The number of amastigotes found per 100 cells, which was determined using both the average amastigotes found per cell and the percentage of infected cells. 200 cells were counted, bars represent ± SEM, each point represents one mouse (n = 9 mice total, 3 mice per experiment). The same statistical test was applied as in A).

After 6 hours, the percentage of cells infected revealed that significantly fewer cells were infected in the *L*. *major*^KO^ group (~17%) compared with *L*. *major*^WT^ and *L*. *major*^R^ (~30%, p<0.05) (Fig 8A). Infection with the GP63 rescue parasites resulted in more cells infected with *L*. *major*. In the observed cells, a higher percentage of neutrophils were infected compared to macrophages, about 30% compared to 10%. The majority of the neutrophils contained 2–3 amastigotes, while macrophages accommodated a higher average number of amastigotes at a time. The presence of GP63 resulted in more parasites found within cells, showing higher infectivity (p<0.05) (Fig 8B). These results indicate that GP63 allows *L*. *major* to more effectively enter host cells.

### *Leishmania* GP63 favour infection progression in macrophages

The cells retrieved from the lavage were seeded on chamber slides and were observed 24 hours and 48 hours post infection. At these time points, the cells were fixed immediately and stained to count the infection progress. At these time points, there were no free leishmania nor neutrophils remaining in the chambers. Consistent with the cytospin infection counts, the *L*. *major*^WT^ and *L*. *major*^R^ parasites resulted in the highest percentage of cells infected (Fig 8C). The percentage of infected cells also increased over time for the *L*. *major*^WT^ and *L*. *major*^R^ infected cells, where at 24hrs *L*. *major*^R^ infected over 3 times more cells than *L*. *major*^KO^ (p<0.001). In the *L*. *major*^KO^ groups, the difference between 6 hours and 12 hours was not as great as that seen in the other groups, where the mean percent of cells infected decreased from 13% to 11%. Between 6 and 24 hours, the mean percentage of *L*. *major*^WT^ infected cells increased from 18% to 25%, and *L*. *major*^R^ infected cells increased from 22% to 39%. The percentage of infected cells increased two-fold at the 48-hour time point for the *L*. *major*^KO^ group, which demonstrates the initial killing of the parasites. Only after the initial infection is the parasites able to infect more cells and re-establish the infection. These numbers were also reflected in the total number of amastigotes found within the infected cells (Fig 8D). Statistical significance was not seen between *L*. *major*^WT^ and *L*. *major*^KO^ groups at 48 hours. These results demonstrate that parasites lacking GP63 are less infective during early infection and that reinsertion of GP63 seems to exacerbate some infectious characteristics conferring greater fitness once within the host cell.

## Discussion

Cutaneous leishmaniasis (CL) caused by *L*. *major* remains a major public health concern to people living in endemic areas, as lesions can be acute or become chronic and slow healing [1]. To this day, knowledge gaps still exist in the early innate immune events in response to *Leishmania* in human hosts. This response is modulated by various *Leishmania* virulence factors to favour parasite survival, which in turn delay clearance and prolong disease. From the past research in our lab, we have shown that GP63 is a significant virulence factor involved in the attenuation of macrophage immune function and signalling [38]. The early moments following *Leishmania* inoculation are extremely crucial for the progression and outcome of disease, but not thoroughly studied. Gaining a better understanding of the major virulence factor GP63 and how it modulates the host immune response globally will provide avenues for the development of better therapies for CL patients.

Previous studies performed in susceptible BALB/c mice using *L*. *major*^KO^ demonstrated that *Leishmania* lacking GP63 were less prone to establish infection in comparison to their wild type counterpart [9]. Studies show that cutaneous lesion formation could be a result of the acute infiltration of macrophages, neutrophils, and eosinophils to the dermis [39], but could also be a direct indicator of parasite burden [40]. The reduced lesion formation in *L*. *major*^KO^ parasites may be due to factors such as increased parasite elimination, delayed host cell infiltration, or inability to cause infection, which we aimed to investigate. In our footpad infection in healing type C57BL/6 mice, we saw footpad thickness infected with *L*. *major* ^WT^ increase at a similar rate to the level reported in the literature [9], reaching the same change of 2mm in footpad thickness in both studies. We also saw a delay in lesion development in the *L*. *major* ^KO^ parasites, but they were able to cause inflammation faster than the previously reported findings, at about 5 weeks. Nonetheless, the reduced infection observed for *L*. *major* ^KO^ infected mice over control groups is similar regardless of the different immune responses from the two mouse models (Balb/c vs C57BL/6). Therefore, this finding serves as a clue to investigate the early immune response, to elucidate the cause of reduced footpad inflammation in *L*. *major*^KO^ infected mice. Even though GP63 is a potent modulator of cell signalling and recruitment, there are other virulence factors such as lipophosphoglycan (LPG), cysteine proteases (CPs), and glycosyl inositol phospholipids (GIPLs) that can influence cell signalling [38, 41]. With this in mind, we sought to investigate the early innate inflammatory response, which may reveal the mechanisms behind the different infection outcomes.

Previous studies showed infection with *L*.*major* in mouse skin pouches caused rapid leukocyte recruitment that peaked at 6 hours post-infection [10]. The intraperitoneal infection model allowed for the greater recruitment of cells and the measurement of cytokines and exosomes released in the supernatant [30]. A limitation of this model of infection would be that it is not the closest representation of natural infection because *L*. *major* causes cutaneous leishmaniasis. Previous knowledge suggested the immunomodulatory action of GP63 should induce greater cell recruitment to support the infection, but 6h post peritoneal infection, the total cell recruitment, and cell types were not found to be significantly different between *L*. *major* ^KO^ and the other groups. Most of the recruited cells were neutrophils, which is in line with reported literature for the initial cell recruitment [42]. Neutrophils are essential in the establishment of *Leishmania* infection since they are needed to shuttle parasites to be phagocytosed by macrophages. There is also macrophage activation from antigenic stimulation in all infection groups, as seen in the flow cytometry data of small peritoneal macrophages [32]. The incoming small peritoneal macrophages are derived from monocytes and dominated the population of existing large peritoneal macrophages in all cases, demonstrating that GP63 is not required to recruit small peritoneal macrophages. Cell recruitment data demonstrates that GP63 is not required for the recruitment of immune cells, which mechanism is likely attributed to other virulence factors and PAMPSs.

To further understand the effect of GP63 depletion in the early responses, the cytokines and chemokines released in the peritoneal cavity were measured. The cytokines IL-6, IL1-ß, and TNF-α were the highest in the *L*. *major* ^WT^ group and surprisingly not recapitulated in the *L*. *major*^R^ group; IL-6 in C57BL/6 mice was reported to be responsible for Th2 responses but not required to control the disease since IL-6 KO mice were able to control infection [43]. On the other hand, TNF-α IFN-γ are crucial in *Leishmania* clearance by working synergistically to increase macrophage killing and NO production [44], but at this early stage of the infection, IFN-γ was at negligible levels in our lavage supernatant in all groups, along with several other cytokines like IL-4. Moreover, the chemokines were expressed at similar levels for all three infection groups regardless of GP63 expression. Past reports described the following chemokines were increased during the initial phase of *L*. *major* infection: KC, IL-8, MIP-2, MIP1a and b, CCL5, CXCL10, and CCL2 [45]. Apart from IL-8, and CCL5, all aforementioned chemokines were expressed in our lavage supernatant, regardless of GP63. In this early time point of 6 hours post-infection, we see that neutrophils are the most important and macrophages have only begun to become activated and recruited as per flow cytometry data. Therefore, the cell signalling modulation by GP63, as shown in past research, is not yet observed at a significant level. Moreover, GP63 is not the sole *Leishmania* factor responsible for the recruitment of leukocytes to the site of infection. *Leishmania* expresses a collection of virulence factors that could affect the inflammatory milieu, for example, cysteine proteases (CP) can degrade NF-κB, STAT-1, and AP-1, similar to the effects of GP63 [38].

By performing transcriptional profiling of the peritoneal cells at 6h post-infection, we also observed a very strong pro-inflammatory response and recruitment of neutrophils, but more importantly, we were also able to detect significant differences between the *L*. *major* ^WT^ and *L*. *major* ^KO^ groups. Interestingly, the transcriptional profile obtained following infection with *L*. *major* ^R^ is not different from the *L*. *major* ^KO^, suggesting that some of the differences between *L*. *major* ^WT^ and *L*. *major* ^R^ parasites could be attributed to GP63 genes 6 and 7, which were not rescued and that may have differences in proteinase substrate specificities [9, 46]. As observed in the cytokine array analysis, infection by either *L*. *major* parasites triggers a similar transcriptional activation for genes encoding cytokines and chemokines. Other cytokines not discussed above have been described to impact the outcome of disease; most notably IL-12 is required for a Th1 driven response for parasite clearance by signalling through the JAK/STAT pathway [47]. IL-12 was seen to be selectively inhibited in inflammatory macrophages infected with *Leishmania* [48], and we know GP63 is an inhibitor of JAK/STAT signalling. Along those lines, we observe that at the transcriptional level, peritoneal cells from *L*. *major* KO/R-infected animals express significantly more *Il12b*. Strikingly, the genes clustering with *Il12b* (cluster #3), more expressed in *L*. *major* KO/R, are known to be involved in Th1 response like *Il12b*, in the cytotoxic cell function and the interferon response pathway. Altogether, the transcriptome analysis suggests that at 6h post-infection, the *Leishmania* GP63 gene cluster can interfere with the activation of key effector and interferon response pathways that are key in the defence against intracellular pathogens.

To further characterize the inflammatory response, the exosomes released were examined because they also play a significant role in intercellular communication. To our knowledge, this is the first reported characterization of total exosomes released by cells in the intraperitoneal cavity following *Leishmania* infection. In this study, we also describe a novel procedure to purify host exosomes from the mouse peritoneal cavity to minimize contamination. International standards for studies in EVs require adequate proof that reports are indeed associated with EVs, therefore we performed NTA, TEM, and proteomic analysis for our samples [49]. The presence of *Leishmania* GP63 seemed to induce more exosome production from recruited cells, although not statistically significant. Studies have shown that cellular stress can induce exosome release as a way to eliminate waste and induce pathological signals to surrounding cells [50]. The autophagy process was also shown to induce exosome release [51], and autophagy was reported to facilitate infection in *Leishmania* infected macrophages [52]. The proteolytic action of GP63 on the cell membrane can cause cellular stress, but more research needs to be done to elucidate the role of GP63 in host exosome secretion. However, it is clear that modulation of phagocytic cells upon infection in vivo could be sufficient to enhance exosomes released in the lumen of the peritoneum. Even with careful extraction and washing, some non-exosome contaminants such as mouse keratin and serum albumin were still detected in the samples; a weakness in this dataset. Gene ontology analyses revealed that biological processes and molecular functions of the protein profiles are similar between all groups, including the PBS group. We also identified several immune related proteins, which showed similar levels between all infection groups. For example, neutrophilic granule protein was found in all infection groups, which is reported in UniprotKB [53] as a protease inhibitor involved in the defence response. Since the cell population found in the intraperitoneal cavity is highly diverse, the inflammatory nature of macrophage exosomes found in vitro [13] is less likely to be observed in our sample.

Lastly, we looked at the level of parasitemia at the time of lavage collection and tracked the infection levels over time. At 6 hours, ~1.6x more neutrophils and macrophages contained amastigotes when infected with *L*. *major* expressing GP63 compared to *L*. *major KO*. As expected, GP63 is a crucial factor for the efficiency of infecting host cells in the early moments of inoculation. GP63 proteins can bind macrophage complement receptors 1 and 3 through the cleavage of C3 to iC3b, as well as interact with receptors B1 integrins [54, 55]. They also degrade the extracellular matrix to favour migration into the cells, increasing the susceptibility in other cell types [5]. We saw the highest number of neutrophils infected because it was the primary cell type that was recruited during the 6 hr infection time frame. Neutrophil recruitment favours the development of leishmaniasis because infected neutrophils act as "trojan horses" to shuttle amastigotes to macrophages [42]. There was a smaller difference in the number of macrophages infected, presumably due to the anti-*Leishmania* activity that GP63 does not attenuate in macrophages. In a previous study using *Leishmania* with GP63 genes 1–6 knockout, Joshi et al. [8] saw no apparent difference between the infectivity of the knockout parasites and wild type when infecting macrophages in vitro. Our findings contradict their results, because the difference of cell interactions in an in vivo infection may more accurately describe the infection in mice.

After the entry into host cells, amastigotes must be able to survive the harsh host environment and be able to replicate and subsequently infect new cells [56]. In hamster peritoneal macrophages, the infectivity and multiplication of *Leishmania* amastigotes were seen to increase over the course of seven days post infection [57]. The survival of the amastigotes internalized by cells was monitored 24 and 48 hrs post-infection in chamber slides and revealed that *L*. *major* KO parasites were less able to survive within the host macrophages and have greater difficulty infecting new cells. There were few free promastigotes found in the lavage; therefore, the number of cells infected is representative of the progression from the 6-hour cell count. Further infection of macrophages is due to the phagocytosis of apoptotic infected neutrophils, as well as replicated amastigotes exiting infected cells [42]. These results corroborate previous knowledge that host anti-*Leishmania* effectors such as NO production, phagolysosome maturation, and ROS inflammasome activation can come into play when not inhibited by GP63 [38, 58]. The surviving cells were then able to infect new cells, as seen at 48 hours post-infection. Our findings confirm previous findings that early elimination of *Leishmania* is crucial in the control of disease [4], where the development of a Th1 response is a result of proper macrophage and NK cell activation [59]. However, our findings also contradict the study with GP63 genes 1–6 knocked out because Joshi et al. observed that GP63 did not affect the survival of amastigotes in macrophages 96 post infection in vitro [8]. They did, however, observe the same delay in footpad infection, suggesting that in vivo interactions with other immune cells are more representative of the infection, which we were able to observe.

Ultimately, our study demonstrated a detailed overview of the innate immune response during the early time points of an L. major infection. The early innate immune response can heavily influence the outcome of disease progression and whether pathology gets controlled. In regard to GP63, it causes a faster and more aggressive lesion development in *L*. *major* cutaneous leishmaniasis. Regardless of GP63, the innate inflammatory response acts similarly in terms of cell recruitment, cytokine/chemokine production, and exosome content. Nevertheless, we see that *L*. *major* KO parasites are less able to infect cells and further infect other cells, most probably because they induce an elevated Th1 and cytotoxic response, as seen in our transcriptome studies. Therefore, the parasite load is reduced, making it more difficult for the surviving *Leishmania* to establish disease. Characterization of factors affecting disease progression and severity can lead to improved treatments for patients with cutaneous leishmaniasis.

## Supporting information

S1 File Raw images for blots and gels. (PDF)

S1 Fig Forward and side scatter of macrophage populations. A) Representative flow cytometry plots of all cell types found in suspension. Sample of PBS and WT is shown to demonstrate an unequal distribution of cell types between different samples. B) Two populations identified in gating for CD11b and F4/80 from intraperitoneal lavages following a 6-hour infection with 10^8^ WT, GP63^KO^, and GP63^R^ *L*. *major* demonstrate significantly different side scatter (SSC) and forward scatter patterns (FSC) which represent the granularity and size of the cells, respectively.
(TIF)

S2 Fig RNA-seq profiles for *L*.*major* strains and *Mus Musculus* peritoneal cells. A) Genes within the GP63 gene cluster are not expressed in GP63^KO^ *L*. *major*. B) More gene examples of genome snapshots are shown in Fig 6.
(TIF)

S3 Fig emPAI values for proteomic analyses of *Mus Musculus* exosomes in the supernatant. Quantitative profiles of protein expression were analyzed using the exponentially modified protein abundance index (emPAI) values. Log10 emPAI values demonstrate the level of up and down regulation of proteins between two groups. Values are sorted by lowest to highest for one group.
(TIF)

S4 Fig Volcano plot for proteomic analyses of *Mus Musculus* exosomes in supernatant. Significant expression of proteins are above the significance threshold line, positive fold change values are higher in the leading group.
(TIF)

S5 Fig UpSet plot for proteomic analyses of *Mus Musculus* exosomes in the supernatant. This is a variation of a Venn diagram to represent exosome proteins found in each group and their intersection. Each row represents a group and the set of proteins found. The grey bars on the left represent the size of the set. Dark circles demonstrate the set is part of the intersection segment in the 4 set Venn diagram. The vertical bars represent the number of proteins found within the particular intersection defined by the circles below.
(TIF)

S6 Fig Gene ontology for proteomic analyses of *Mus Musculus* exosomes in the supernatant. Gene ontology of total proteins is categorized by biological process, cellular compartment, and molecular function. A list of proteins expressed in each group was generated using scaffold (minimum 2 spectrum counts in one sample). The number of proteins in each category is graphed as a percentage of total proteins mapped.
(TIF)

S1 Table Proteomic analysis: Spectrum count, peptide count, exosomes markers, and proteins of interest. (XLSX)

S2 Table RNA-seq analysis of peritoneal cells recovered 6h following PBS injection (control) or *Leishmania* infection with wild-type, GP63 KO, or GP63 rescue *L*. *major*. (XLSX)

S3 Table Gene ontology enrichment analysis for gene significantly dysregulated in peritoneal cells recovered 6h following infection with Leishmania major (wild-type, GP63 KO, or GP63 rescue). (XLSX)
