# Supplementary material for: The role of Leishmania GP63 in the modulation of innate inflammatory response to Leishmania major infection
Source: PLoS One. 2021 Dec 31;16(12):e0262158. doi: 10.1371/journal.pone.0262158 (PMC8719666; doi:10.1371/journal.pone.0262158)
Supplement: S1 File — (PDF) [file pone.0262158.s001.pdf]

Figure 1: Anti- GP63

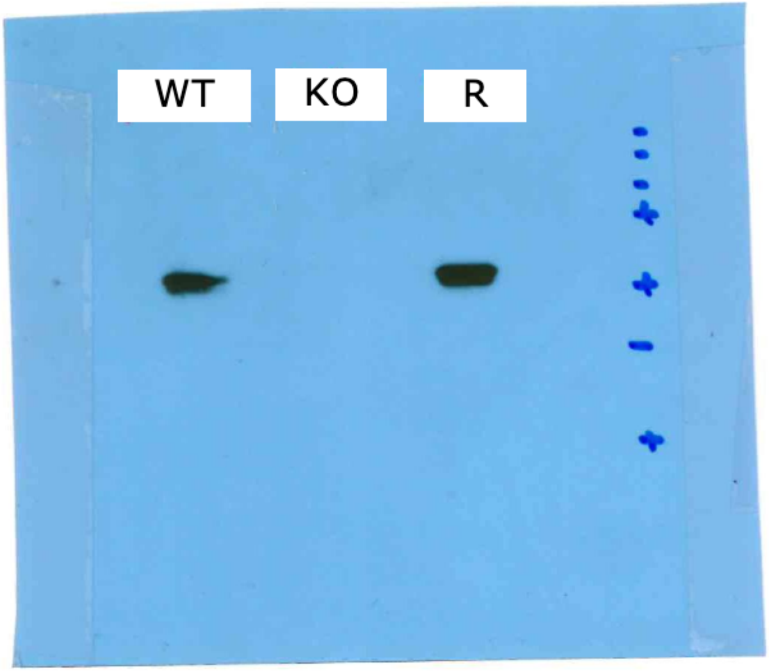

Figure 1: Anti- α tubulin

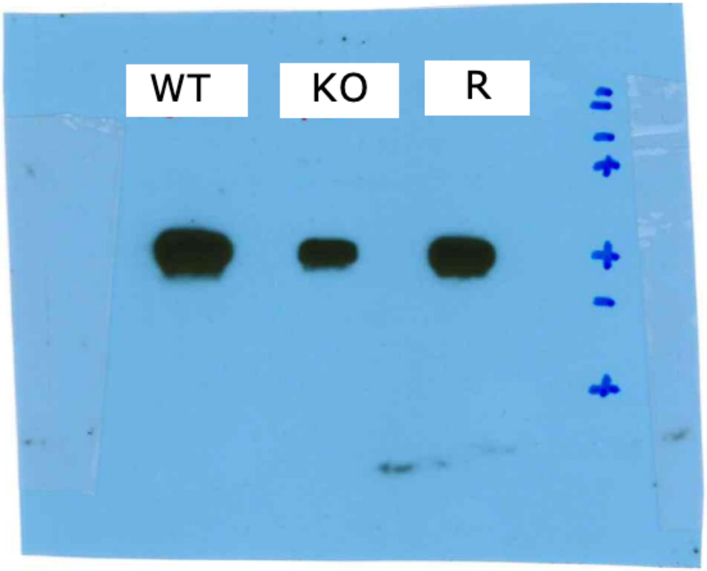

Western blot photos obtained via direct colour scan of x-ray film

Figure 1: Protease activity

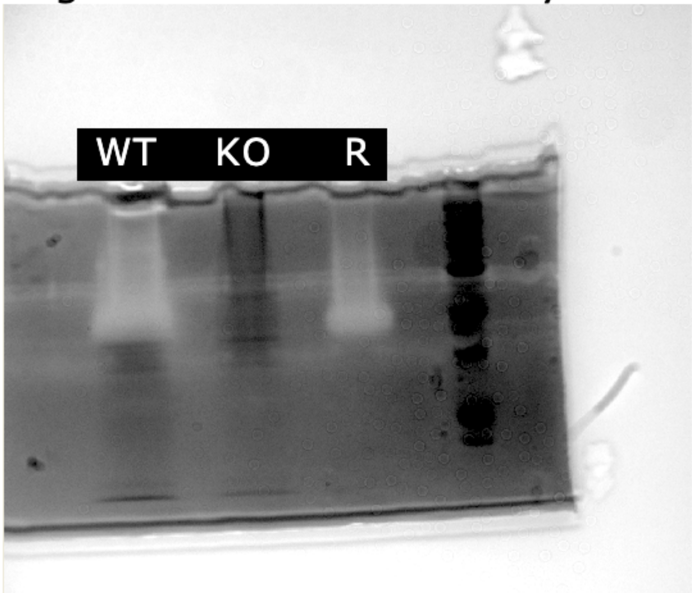

Gelatin zymography photo obtained via GelDoc capture on grayscale setting
