# Supplementary figures and images for: The role of Leishmania GP63 in the modulation of innate inflammatory response to Leishmania major infection
Source: PLoS One. 2021 Dec 31;16(12):e0262158. doi: 10.1371/journal.pone.0262158 (PMC8719666; doi:10.1371/journal.pone.0262158)

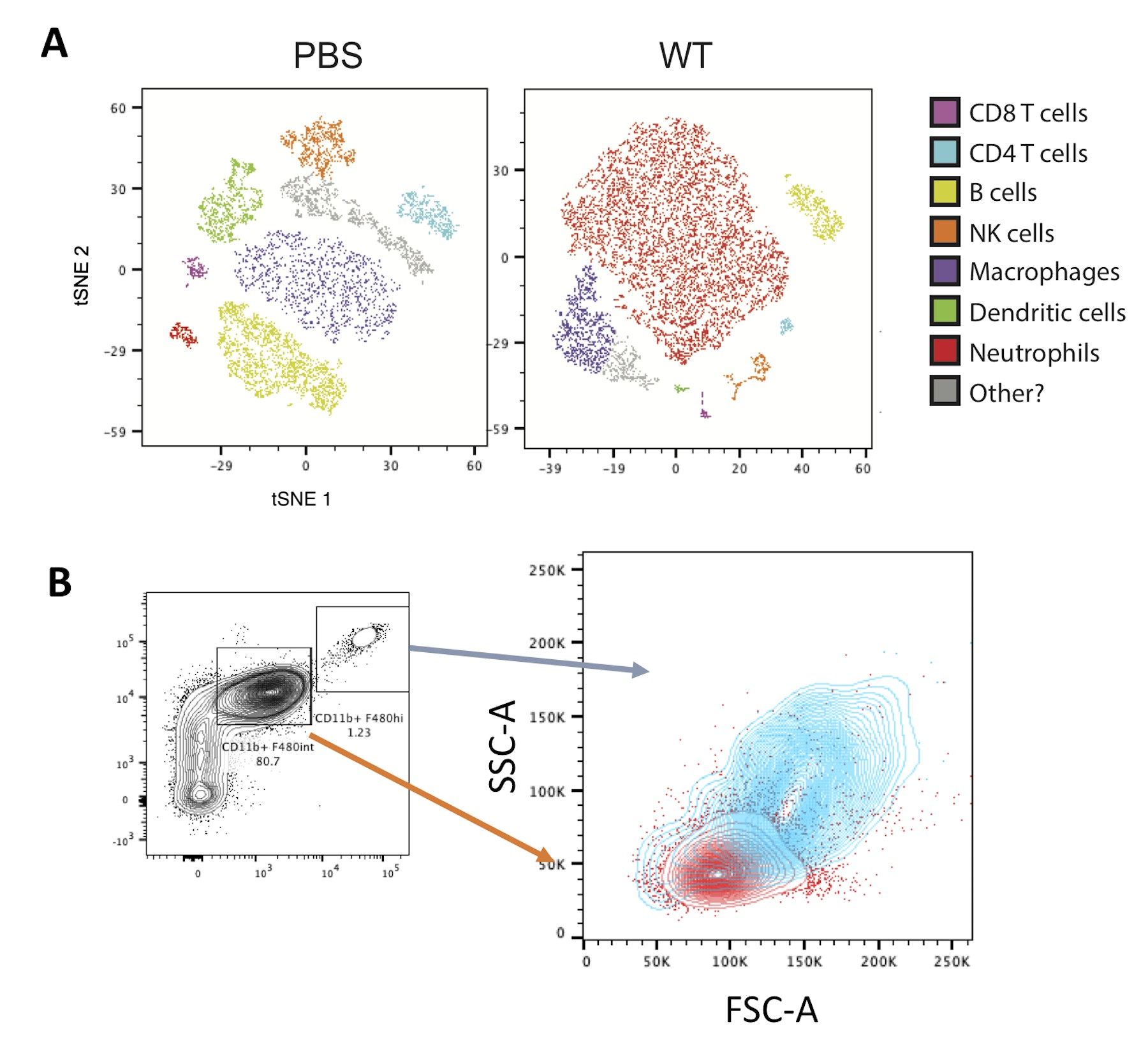

Supplement: S1 Fig — A) Representative flow cytometry plots of all cell types found in suspension. Sample of PBS and WT is shown to demonstrate an unequal distribution of cell types between different samples. B) Two populations identified in gating for CD11b and F4/80 from intraperitoneal lavages following a 6-hour infection with 108 WT, GP63KO, and GP63R L. major demonstrate significantly different side scatter (SSC) and forward scatter patterns (FSC) which represent the granularity and size of the cells, respectively. (TIF) [file pone.0262158.s002.tif]

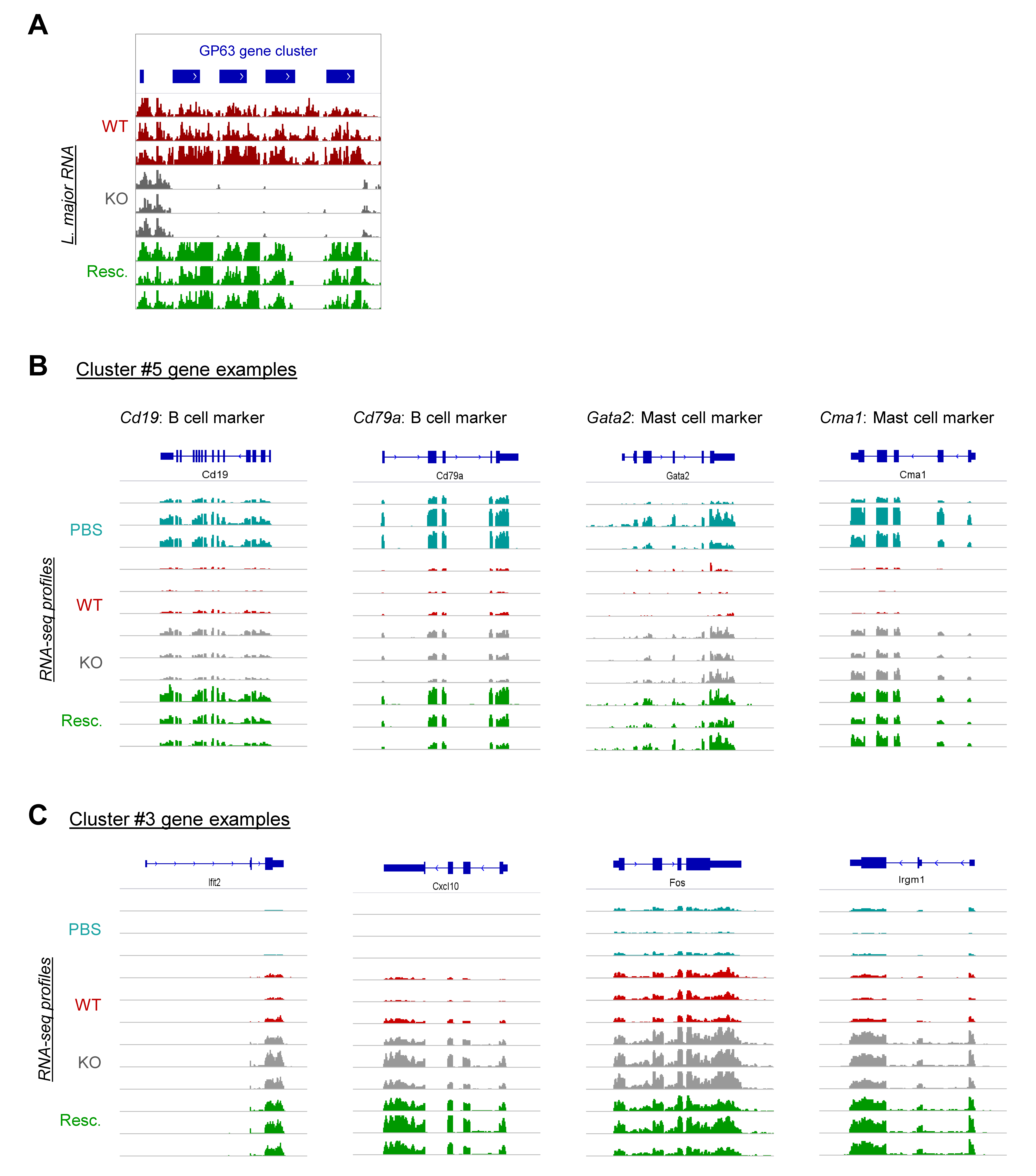

Supplement: S2 Fig — A) Genes within the GP63 gene cluster are not expressed in GP63KO L. major. B) More gene examples of genome snapshots are shown in Fig 6. (TIF) [file pone.0262158.s003.tif]

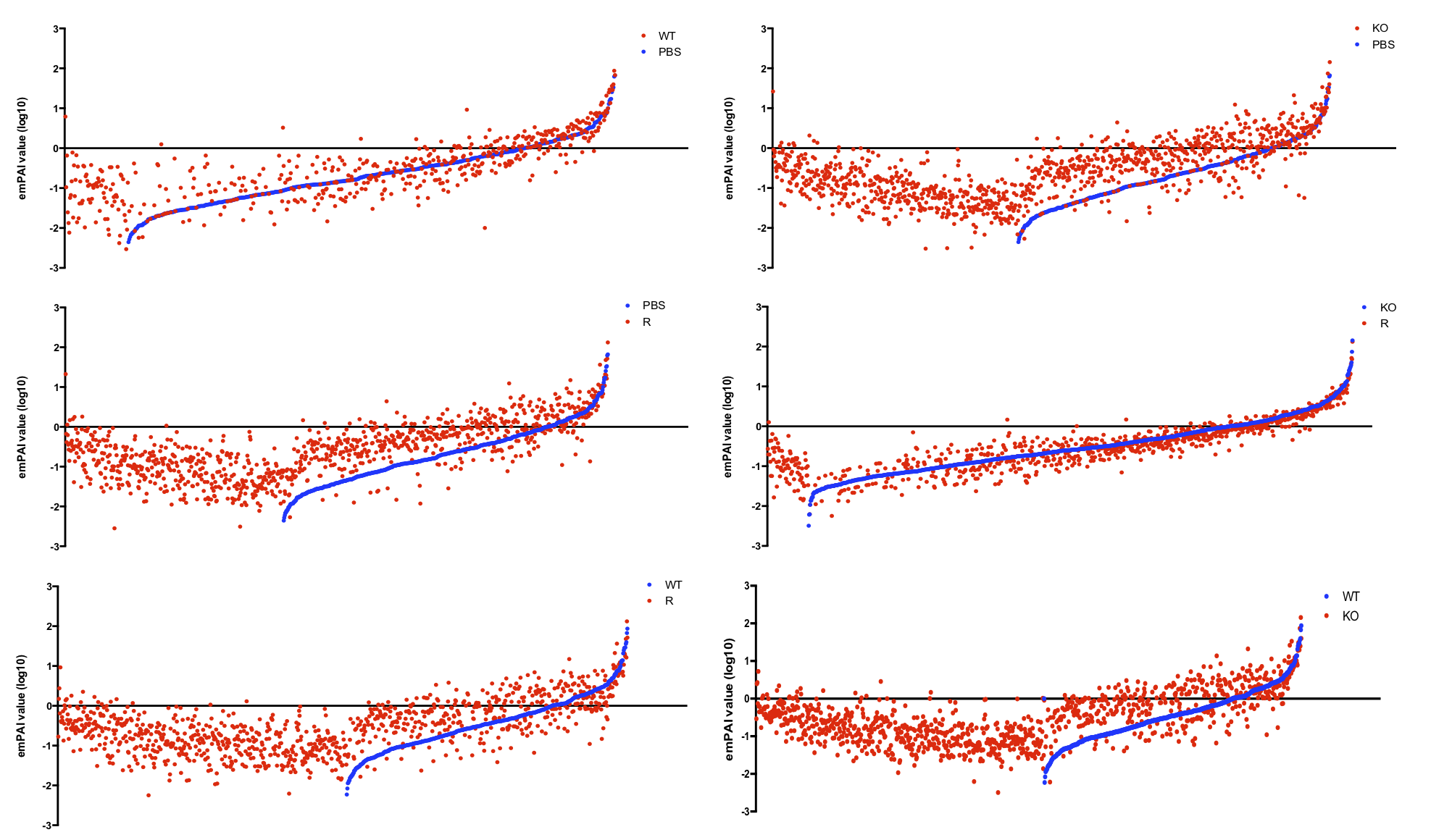

Supplement: S3 Fig — Quantitative profiles of protein expression were analyzed using the exponentially modified protein abundance index (emPAI) values. Log10 emPAI values demonstrate the level of up and down regulation of proteins between two groups. Values are sorted by lowest to highest for one group. (TIF) [file pone.0262158.s004.tif]

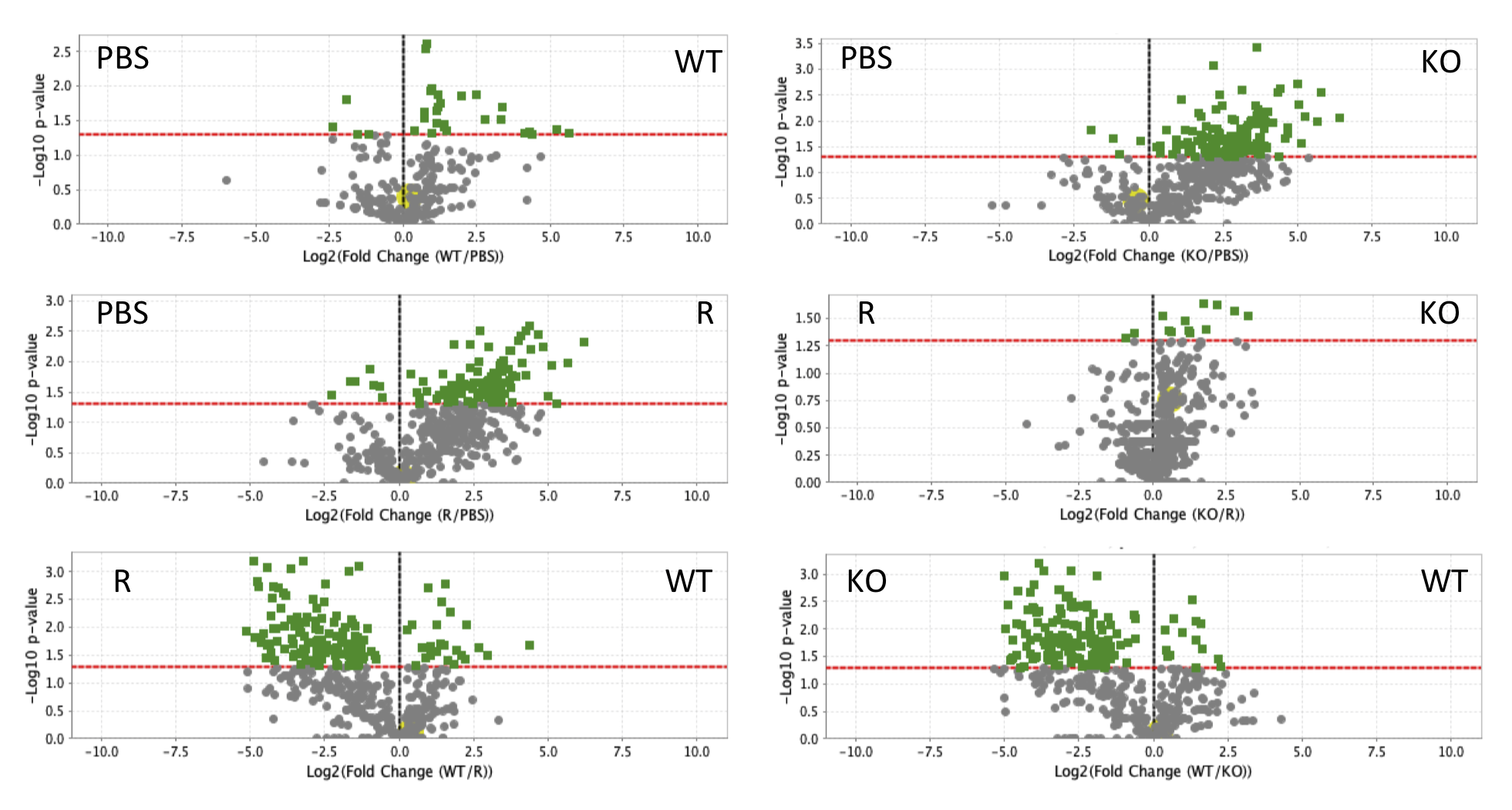

Supplement: S4 Fig — Significant expression of proteins are above the significance threshold line, positive fold change values are higher in the leading group. (TIF) [file pone.0262158.s005.tif]

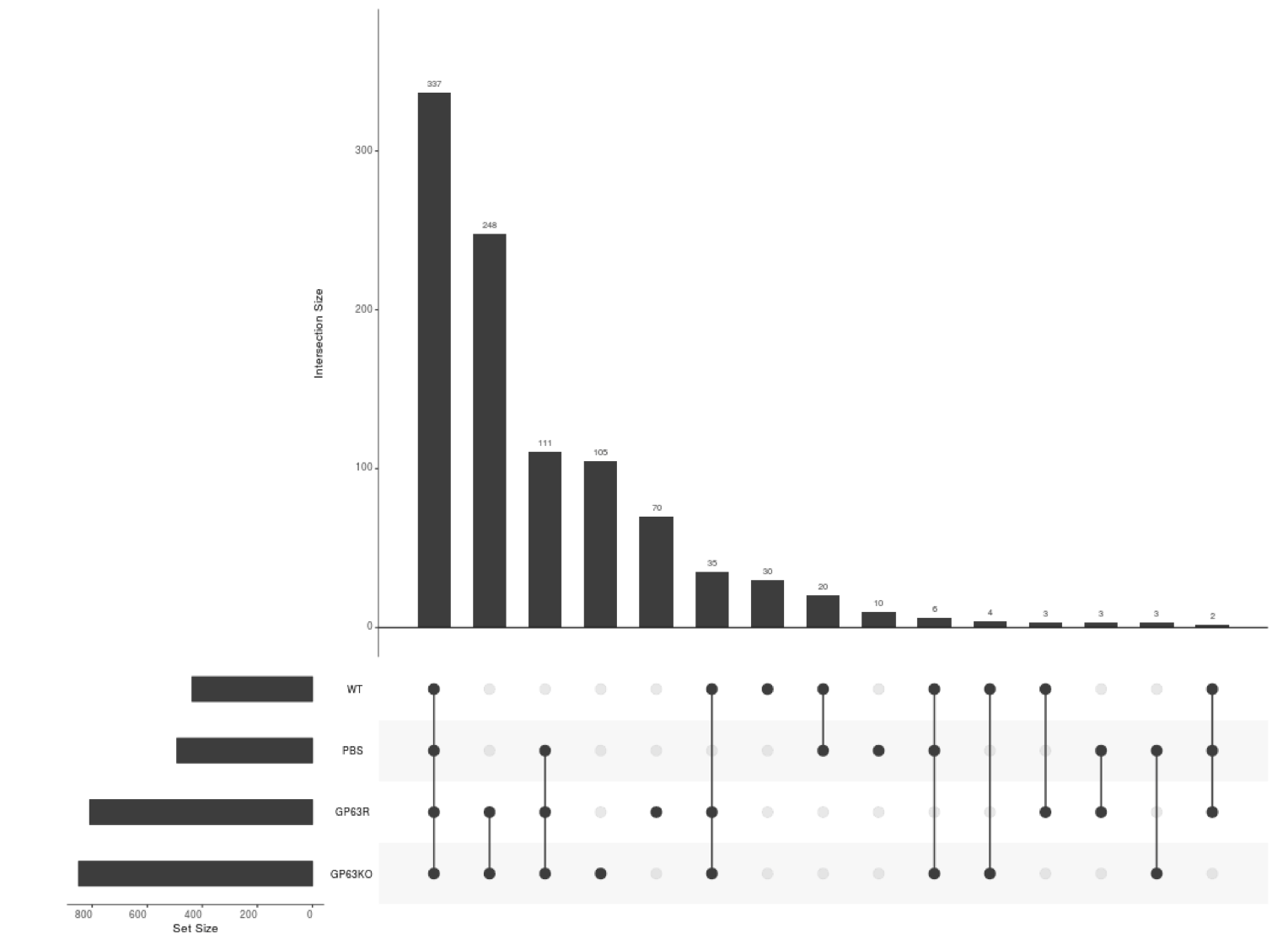

Supplement: S5 Fig — This is a variation of a Venn diagram to represent exosome proteins found in each group and their intersection. Each row represents a group and the set of proteins found. The grey bars on the left represent the size of the set. Dark circles demonstrate the set is part of the intersection segment in the 4 set Venn diagram. The vertical bars represent the number of proteins found within the particular intersection defined by the circles below. (TIF) [file pone.0262158.s006.tif]

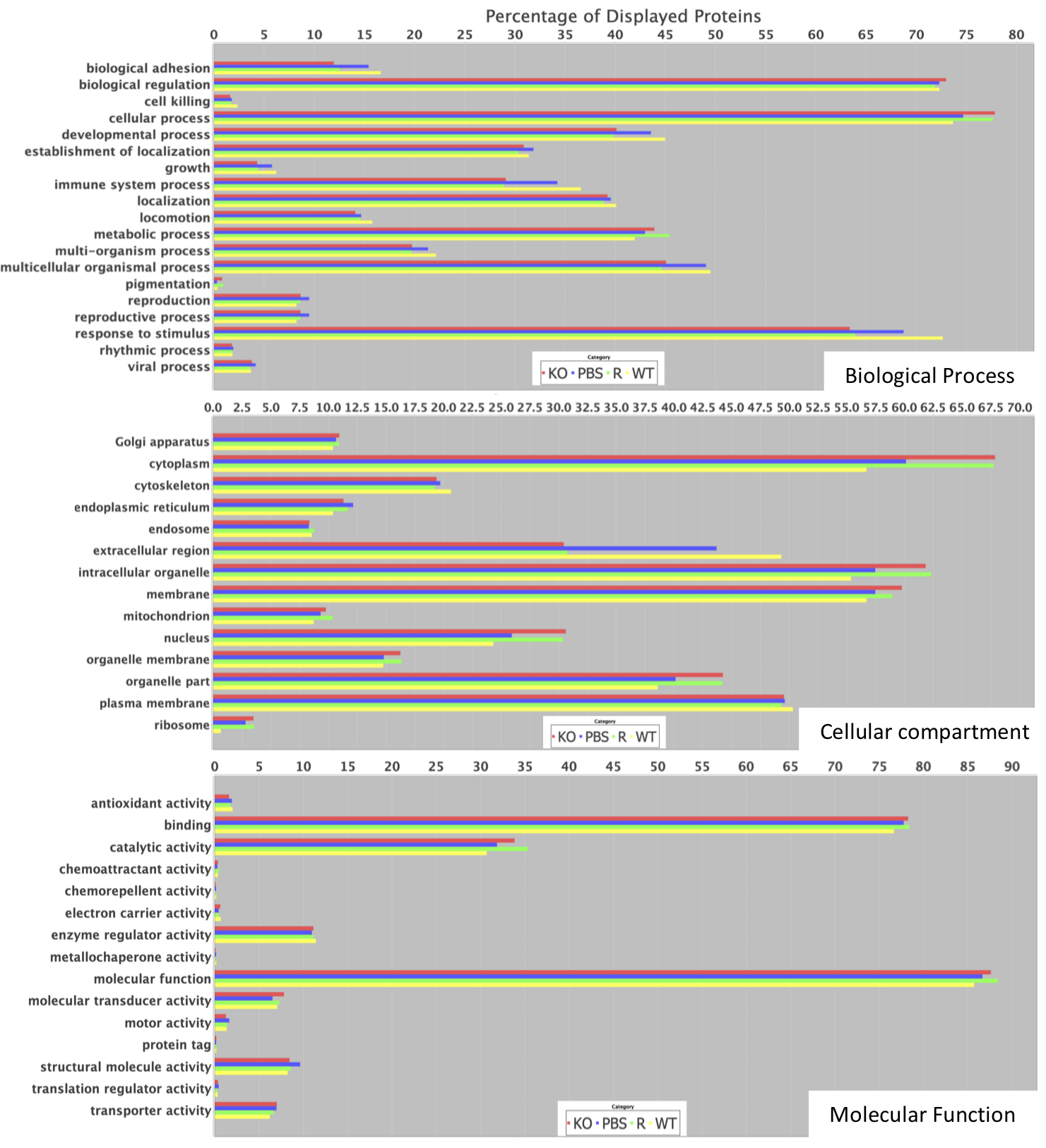

Supplement: S6 Fig — Gene ontology of total proteins is categorized by biological process, cellular compartment, and molecular function. A list of proteins expressed in each group was generated using scaffold (minimum 2 spectrum counts in one sample). The number of proteins in each category is graphed as a percentage of total proteins mapped. (TIF) [file pone.0262158.s007.tif]
